# Supplementary figures and images for: Modeling Toxoplasma gondii-gut early interactions using a human microphysiological system
Source: PLoS Negl Trop Dis. 2025 Feb 4;19(2):e0012855. doi: 10.1371/journal.pntd.0012855 (PMC12136440; doi:10.1371/journal.pntd.0012855)

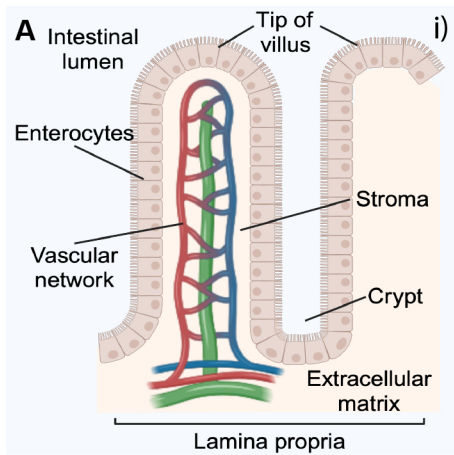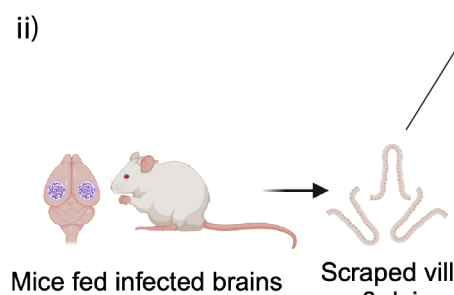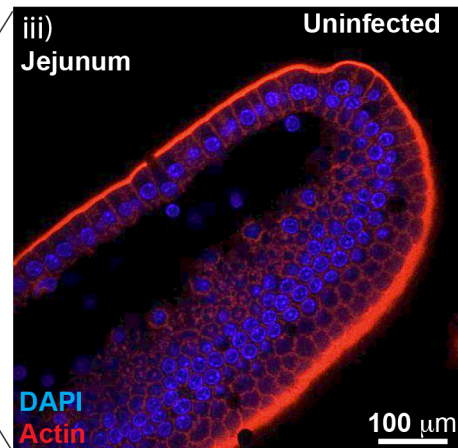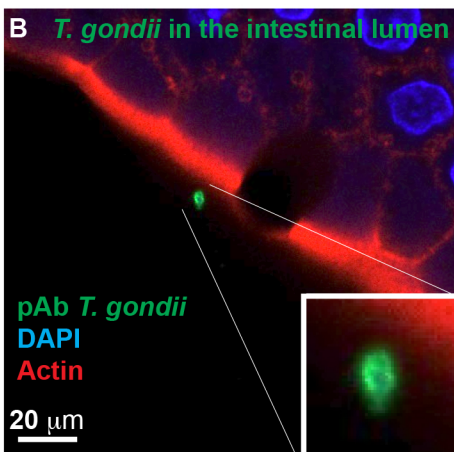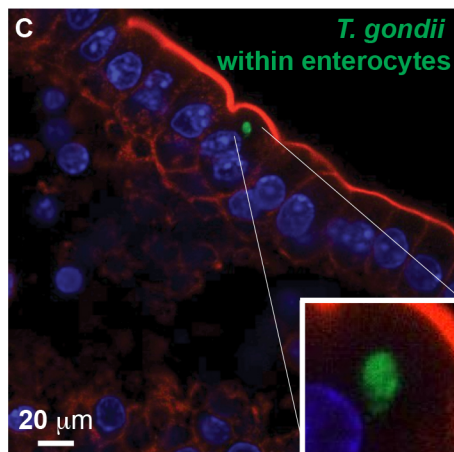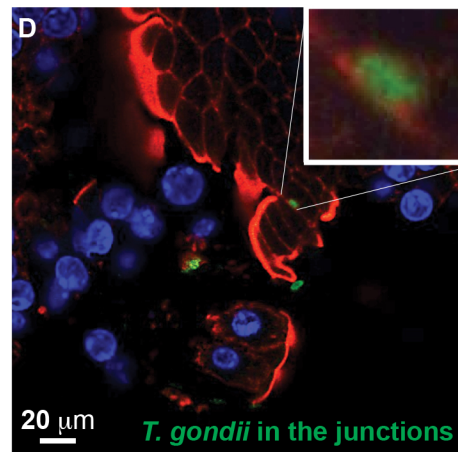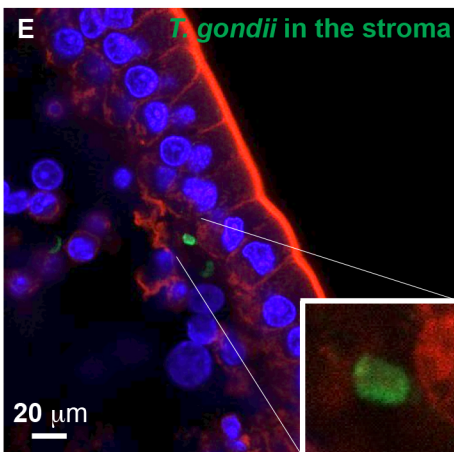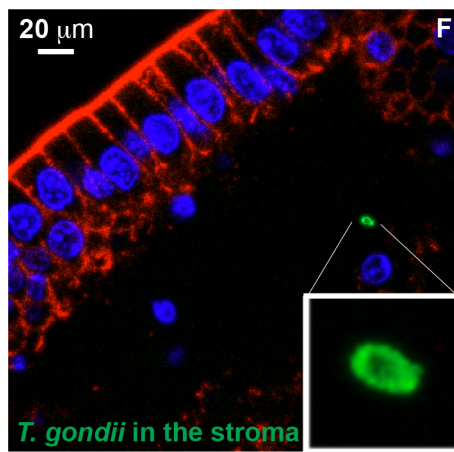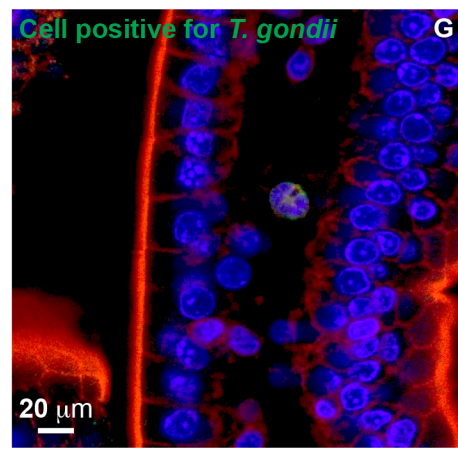

Supplement: S1 Fig — All images represent single events captured in independent infected mice. (A) Schematic representation of (i) the villus and its structures and (ii) the experimental setup; and iii) a confocal image of an uninfected jejunal. (B-G) Confocal images of infected villus with T. gondii after 3 days post-ingestion of cysts-containing brains. T. gondii is localized in the intestinal lumen (B), inside an enterocyte (C), between cell-to-cell junctions (D), in the jejunal stroma (E, F), and within a potential immune cell (G). In all images (A-G), villi are stained for actin (red, rhodamine phalloidin), T. gondii (green, polyclonal antibody), and nuclei (blue, DAPI). Schematic representation in (A) was created with BioRender.com. (PDF) [file pntd.0012855.s001.pdf]

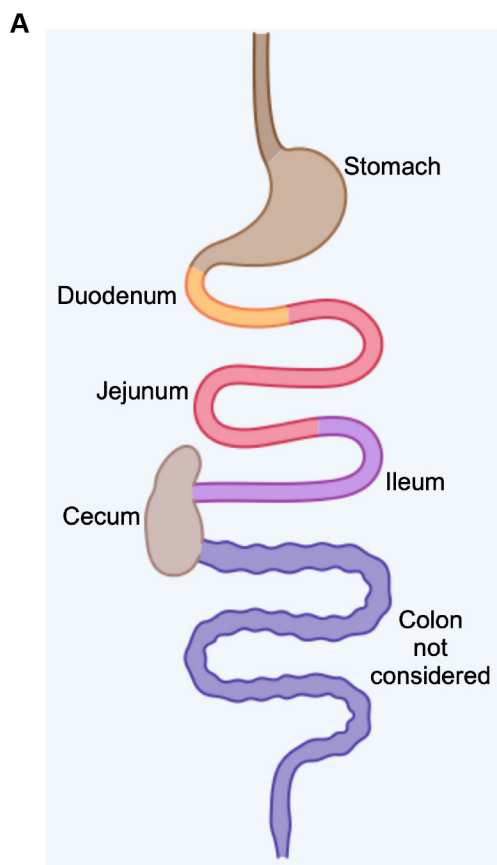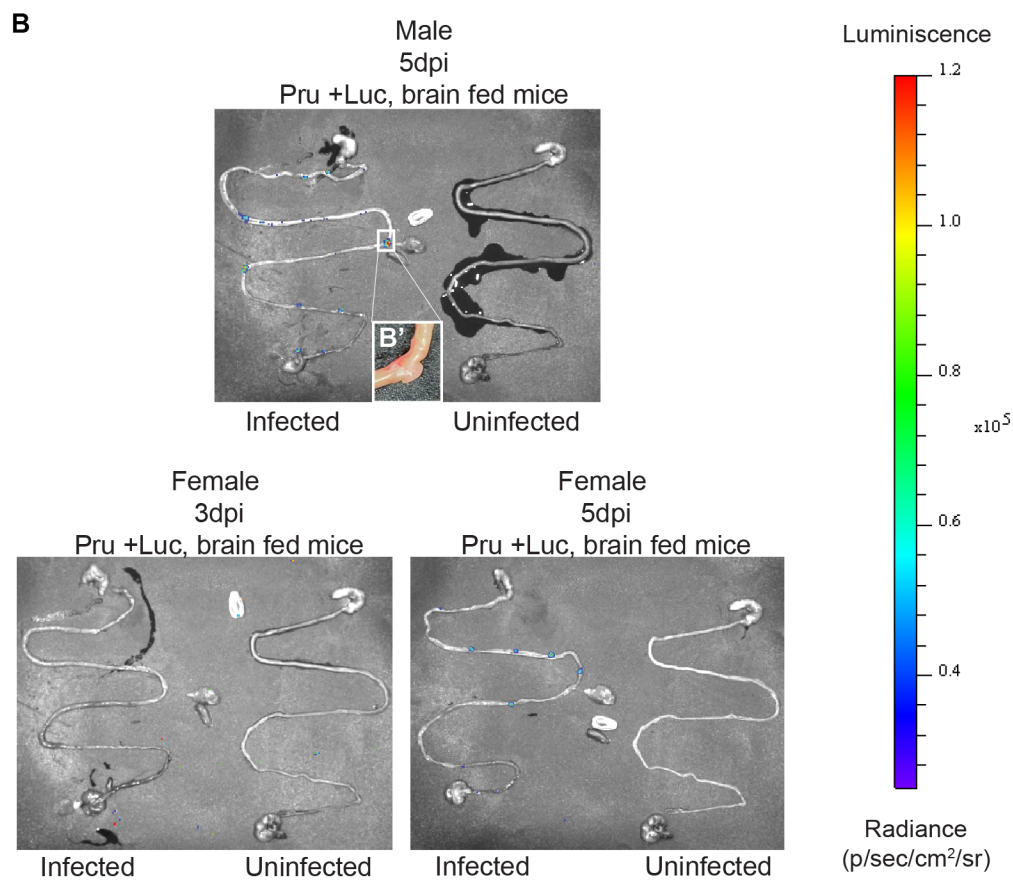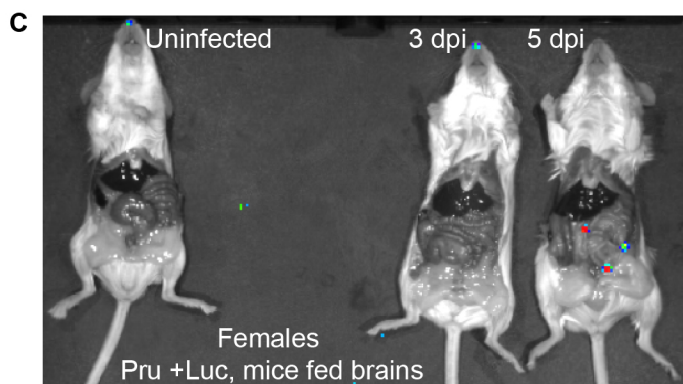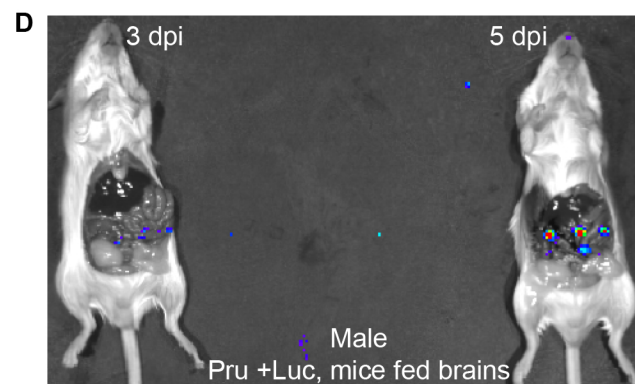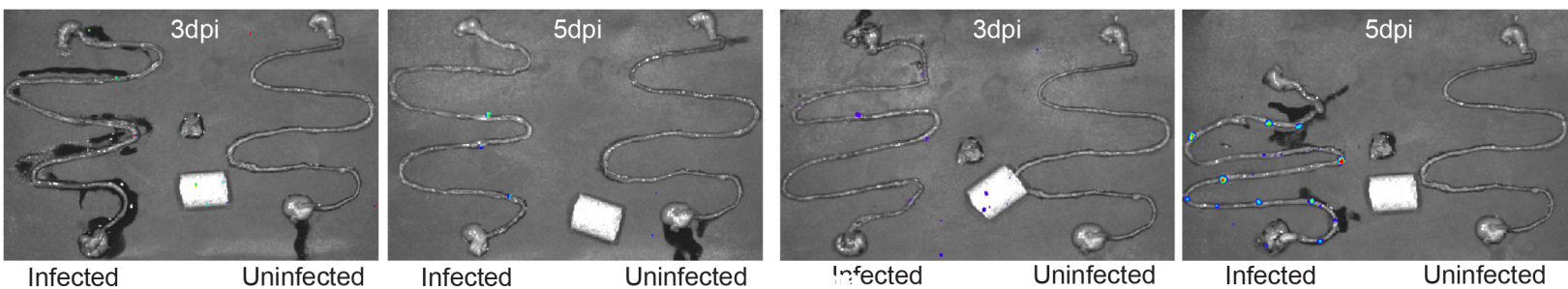

Supplement: S2 Fig — (A) Schematic representation of mice intestine that was considered for the study. (B) IVIS images of one male and one female mouse fed brains containing cysts at 5 days post-ingestion, and one female after 3 days post-ingestion. Mice were fed with sunflower seeds to avoid an unexpected background. B’ inset in the top figure shows a bumped area of the intestine that corresponds with the bioluminescent region detected by IVIS. (C) An uninfected mouse and female mice fed brains containing cysts and small intestine after 3- or 5-days post-ingestion. (D) An uninfected mouse and male mice fed brains containing cysts and small intestine after 3- or 5-days post-ingestion. Schematic representation in (A) was created with BioRender.com. (PDF) [file pntd.0012855.s002.pdf]

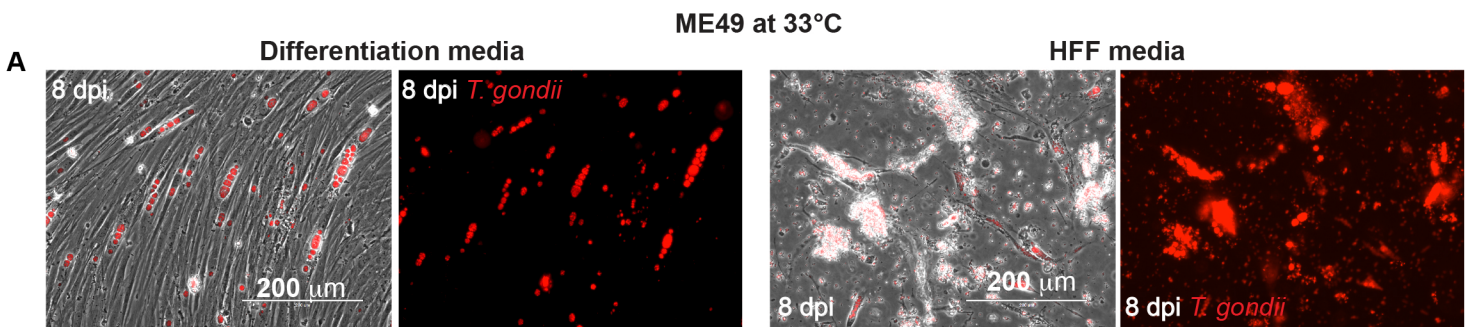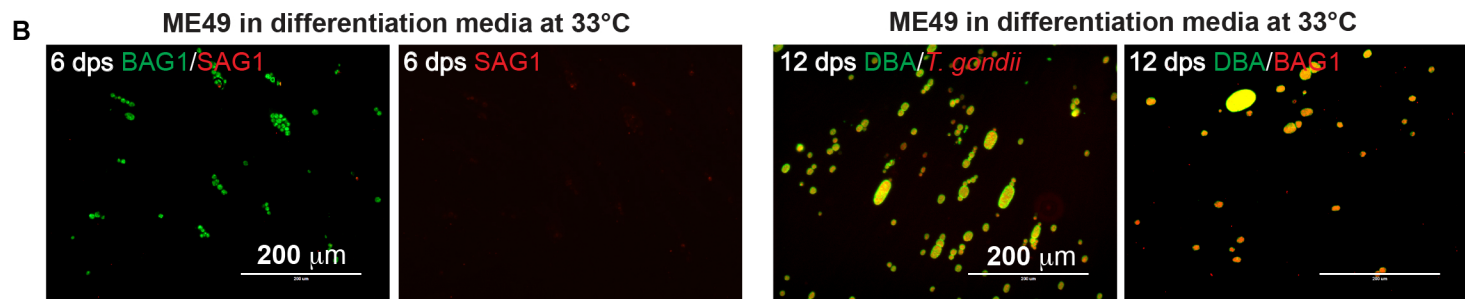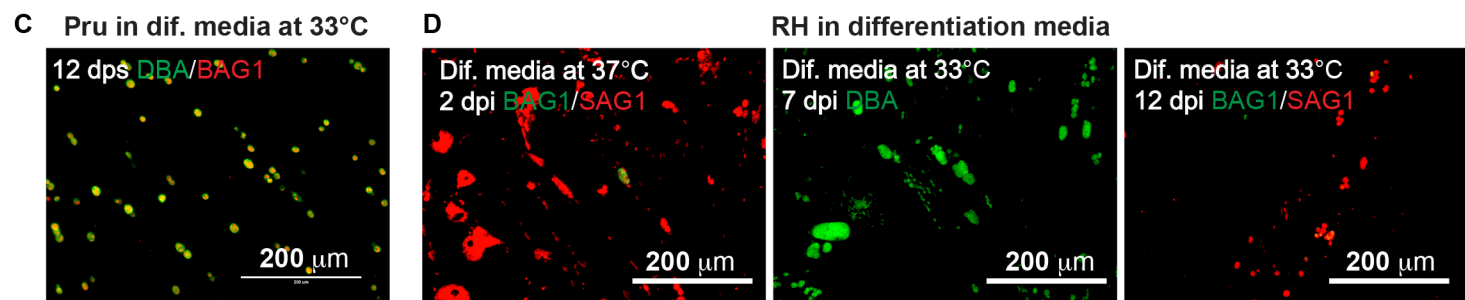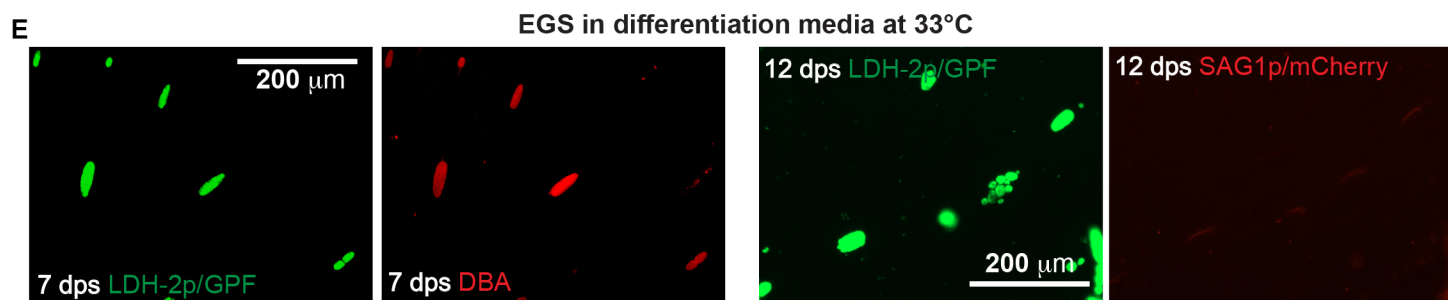

**F** **ME49 mCherry parasites after 72 hpi as bradyzoites**

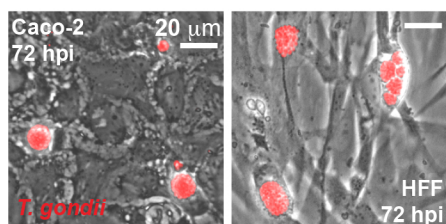

**G**

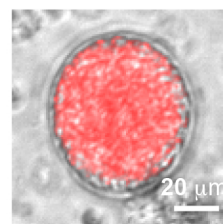

Supplement: S6 Fig — (A) Fluorescent imaging of HFF cells infected with ME49 mCherry for 8-days post-infection in HFF media or differentiation media. (B) Fluorescent images of DBA-, BAG1-, SAG1-positive parasites/cysts after 6- or 12-days post-switching to differentiation media. (C) Fluorescent images of DBA- or BAG1-positive Pru parasites/cysts after 12-days post-switching to differentiation media. (D) Fluorescent images of DBA-, BAG1-, SAG1-positive RH parasites/cysts after 2-, 7- and 12-days post-switching to differentiation media. (E) Fluorescent images of LDH-2p/GFP, SAG1/mCherry or DBA-positive parasites/cysts after 7- or 12-days post-switching to differentiation media. (F) Fluorescent images of Caco-2 and HFF infected with in vitro-differentiated bradyzoites at 72 hours post-infection. (G) Representative image of a brain cyst isolated from an infected mice fed with in vitro-generated bradyzoites after 28 days post-feeding. (PDF) [file pntd.0012855.s006.pdf]

# Growth of EGS at 37°C in differentiation media

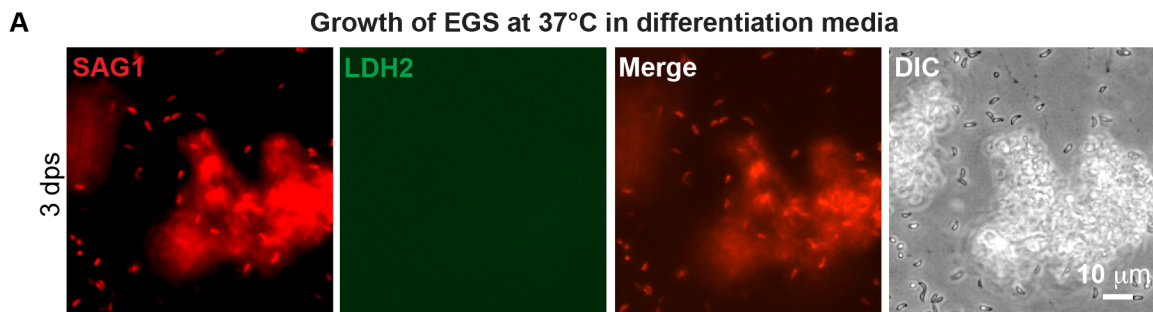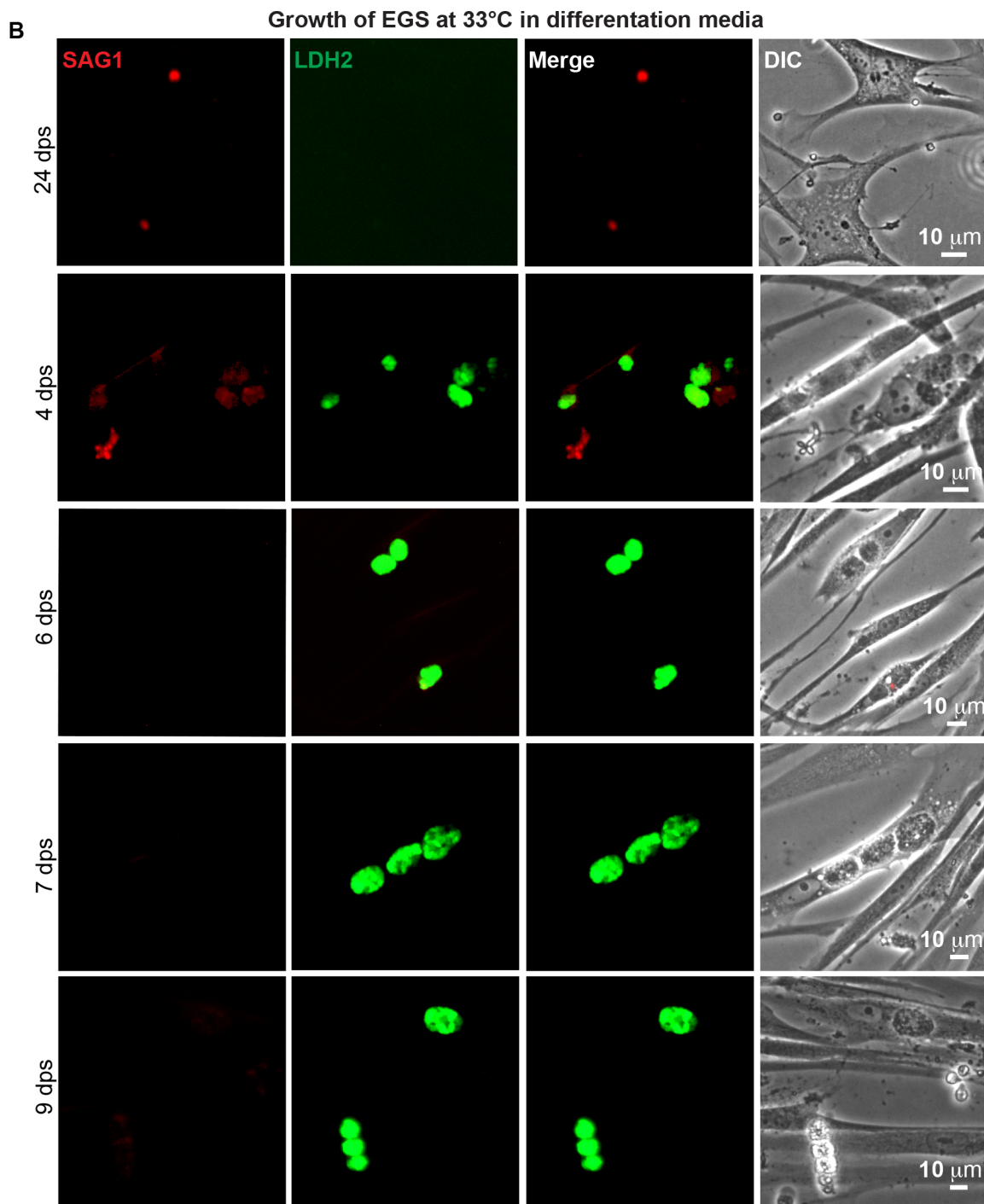

Supplement: S7 Fig — Alive fluorescent imaging of EGS parasites detecting mCherry-expressing tachyzoites or GFP-expressing bradyzoites. (A) Representative fluorescent images of HFF cells infected with EGS parasites at 3 days in differentiation media at 37°C, showing mCherry-expressing tachyzoites. (B) Representative fluorescent images of HFF cells tracking the differentiation of EGS parasites into GFP-expressing bradyzoites under our differentiation protocol at 33°C from one to nine days post-infection. (PDF) [file pntd.0012855.s007.pdf]

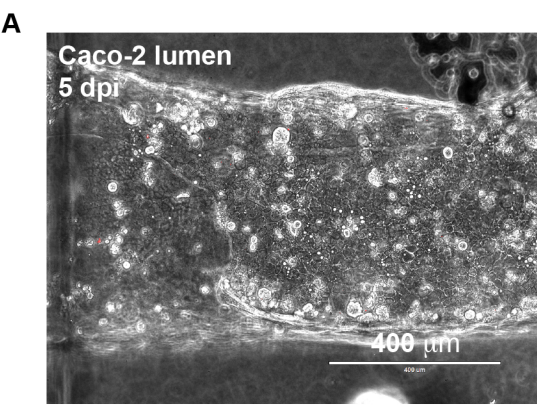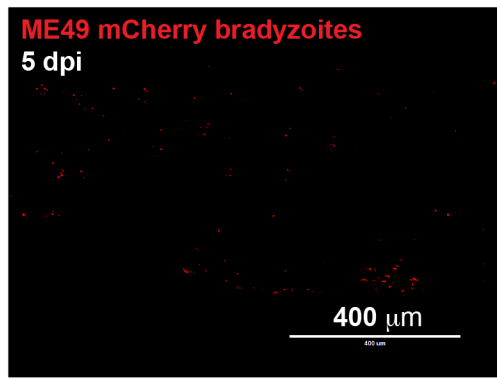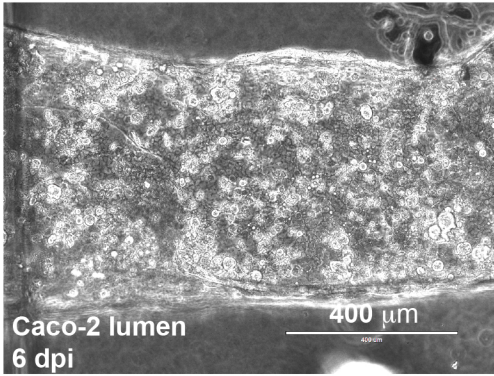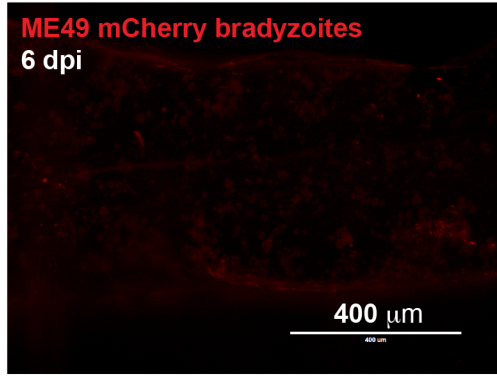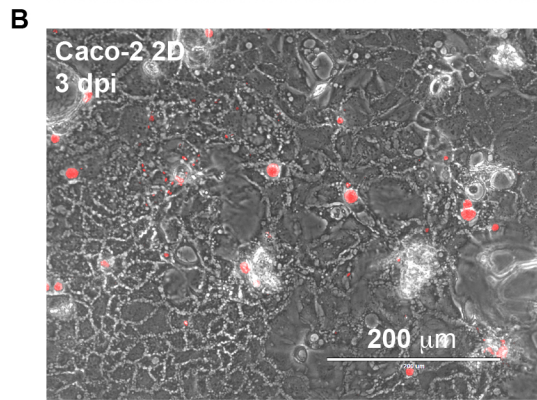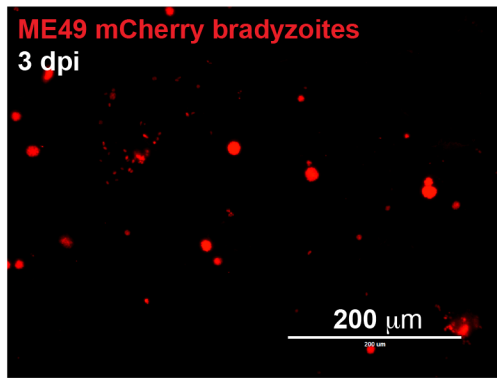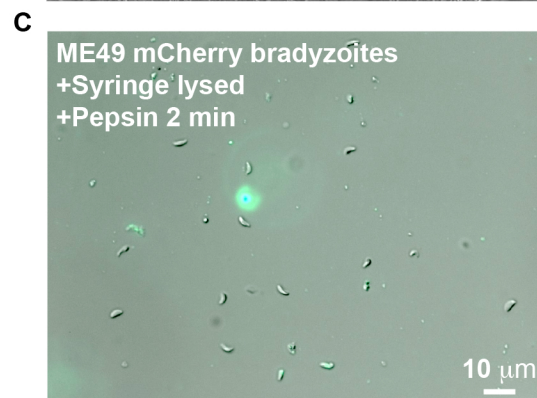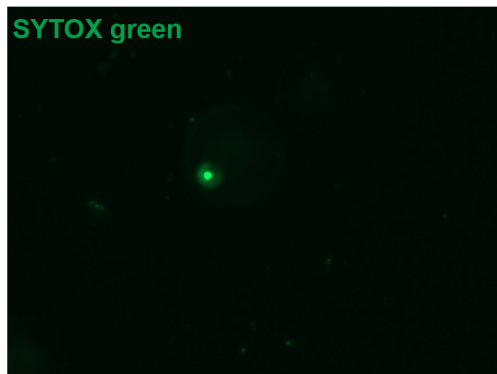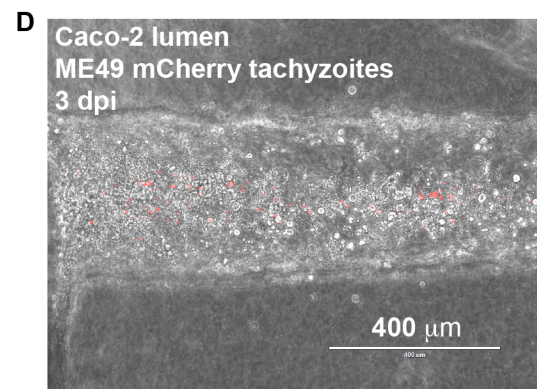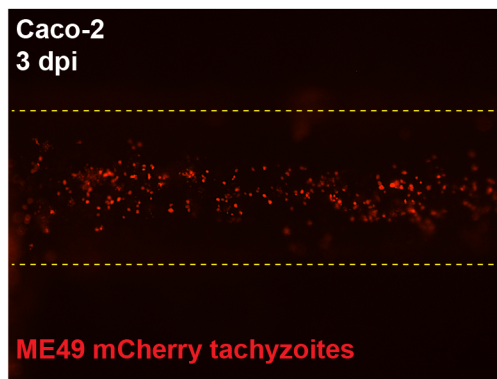

Supplement: S8 Fig — (A) Fluorescent images showing no replication of in vitro differentiated ME49 mCherry bradyzoites in the Caco-2 lumen after 5- or 6- days post-infection. (B) Fluorescent images of ME49 mCherry parasites replicating in Caco-2 after 3 days post-infection, as a positive control of parasite viability. They infected as in vitro differentiated bradyzoites in MPS. (C) Determination of cell viability of in vitro differentiated bradyzoites after pepsin digestion by using SYTOX-green. (D) Caco-2 lumens infected with ME49 mCherry tachyzoites used as a control of infection. Punctuated lines represent the edges of the lumen and its boundary with the matrix. (PDF) [file pntd.0012855.s008.pdf]

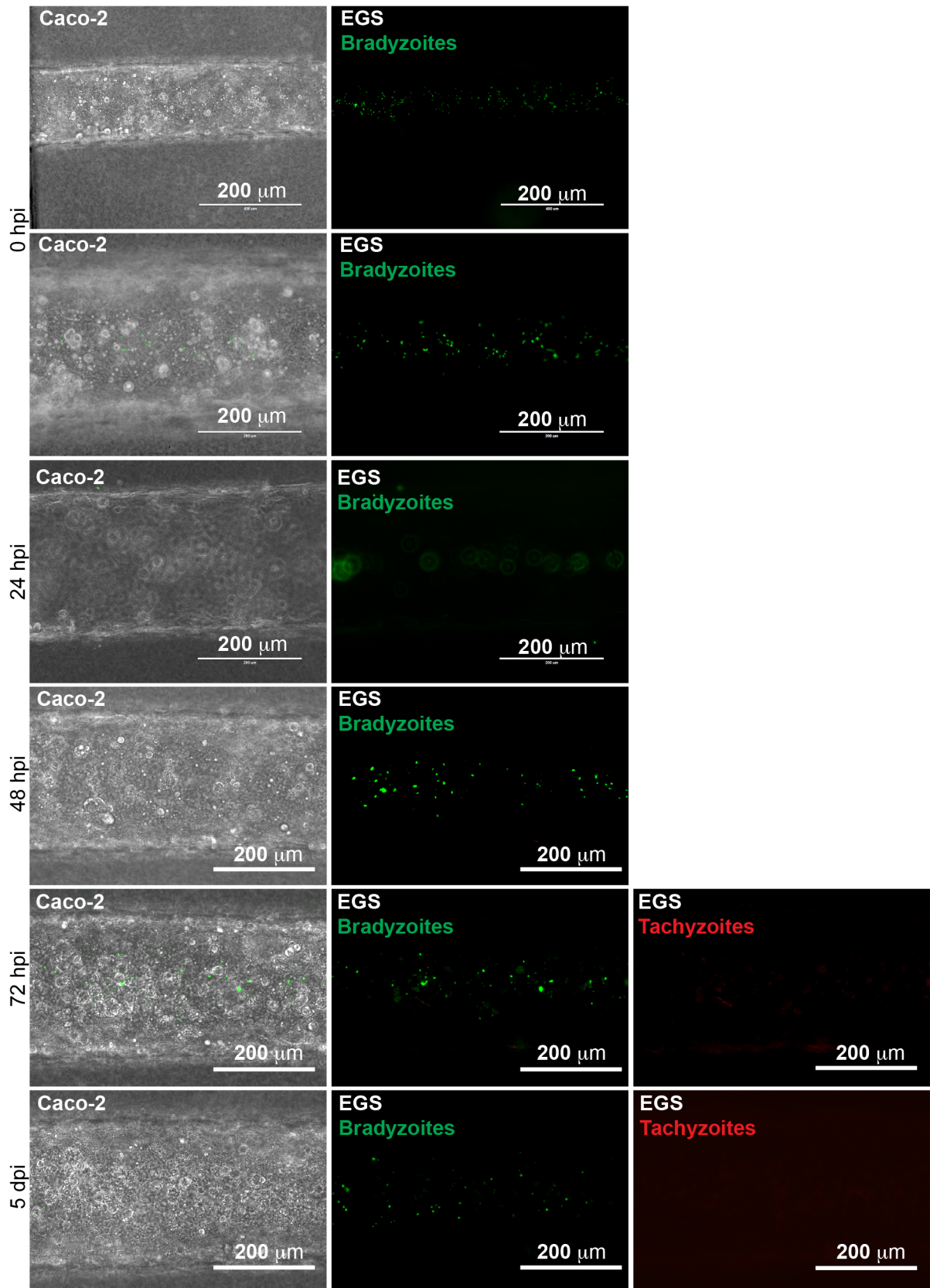

Supplement: S9 Fig — Fluorescent images showing no replication of EGS in vitro differentiated bradyzoites in the Caco-2 lumen after up to 5- days post-infection. (PDF) [file pntd.0012855.s009.pdf]

**A**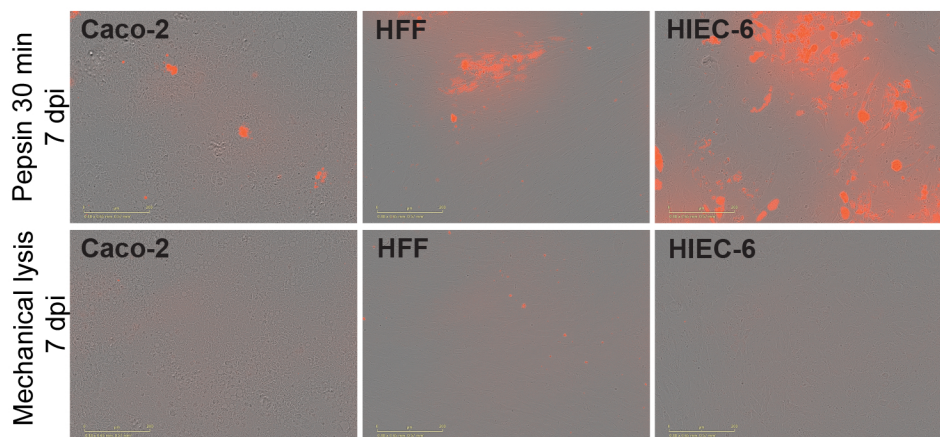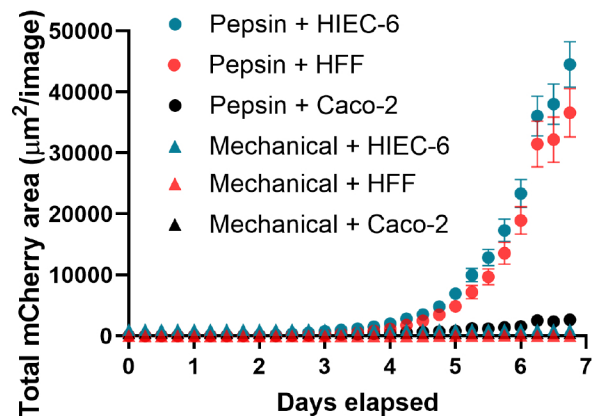**B**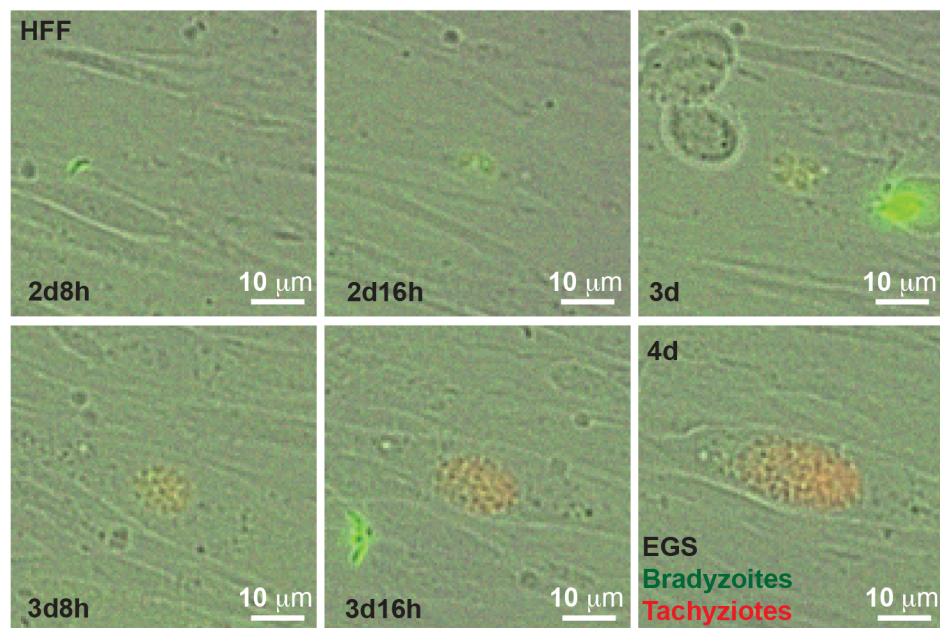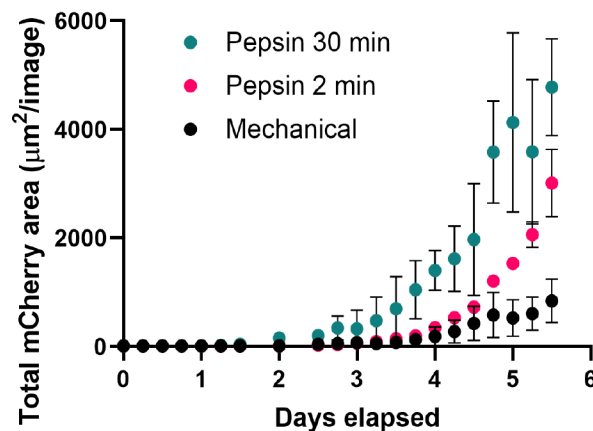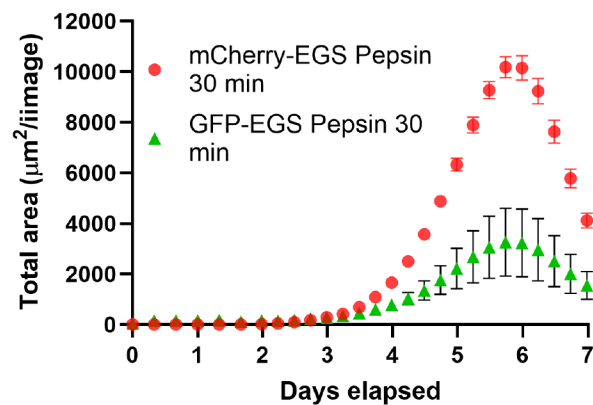

Supplement: S10 Fig — (A) Representative fluorescent images of parasite replication in HFF, Caco-2, and HIEC-6 cells infected with ME49 mCherry brain bradyzoites, either activated by pepsin for 30 min or subjected to mechanical lysis, at 7 days post-infection. Graph shows parasite kinetics of growth in HFF, Caco-2 and HIEC-6 cells for pepsin-activated versus inactivated bradyzoites. (B) Representative fluorescent images of tachyzoite conversion in HFF infected with EGS in vitro differentiated bradyzoites activated by pepsin for 30 min. Conversion and replication were followed up to 4 days post-infection. Top graph shows parasite kinetics of growth in the HFFs cells activated by pepsin for 2 or 30 minutes or inactivated. Bottom graph shows EGS bradyzoite conversion to tachyzoites kinetics in the HFFs cells activated by pepsin for 30 minutes. (PDF) [file pntd.0012855.s010.pdf]

**A**

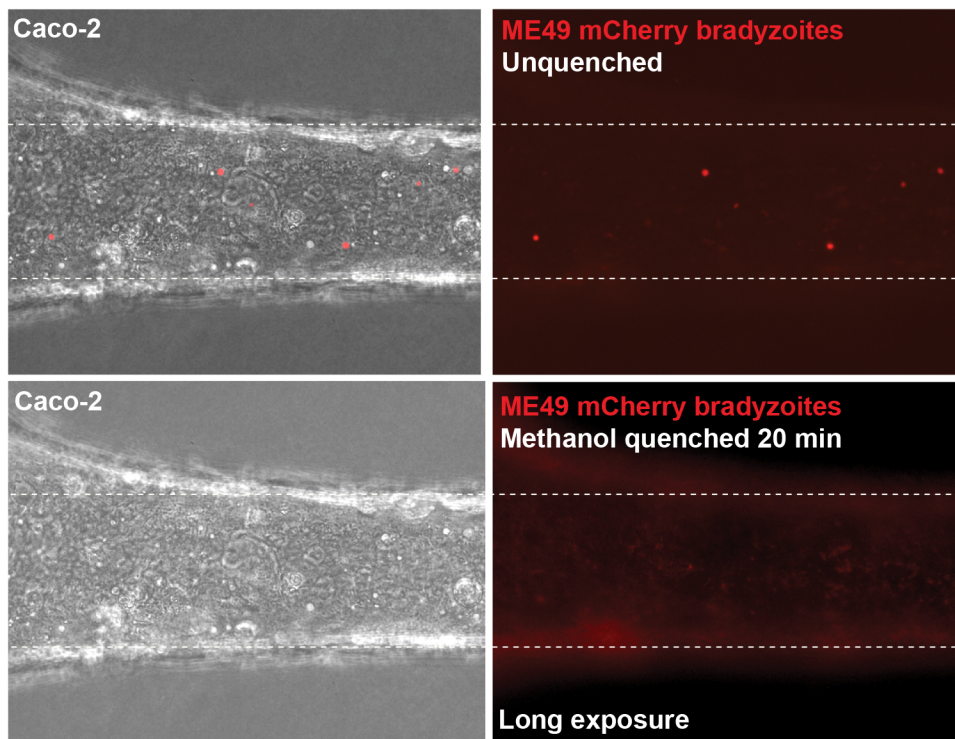

**B**

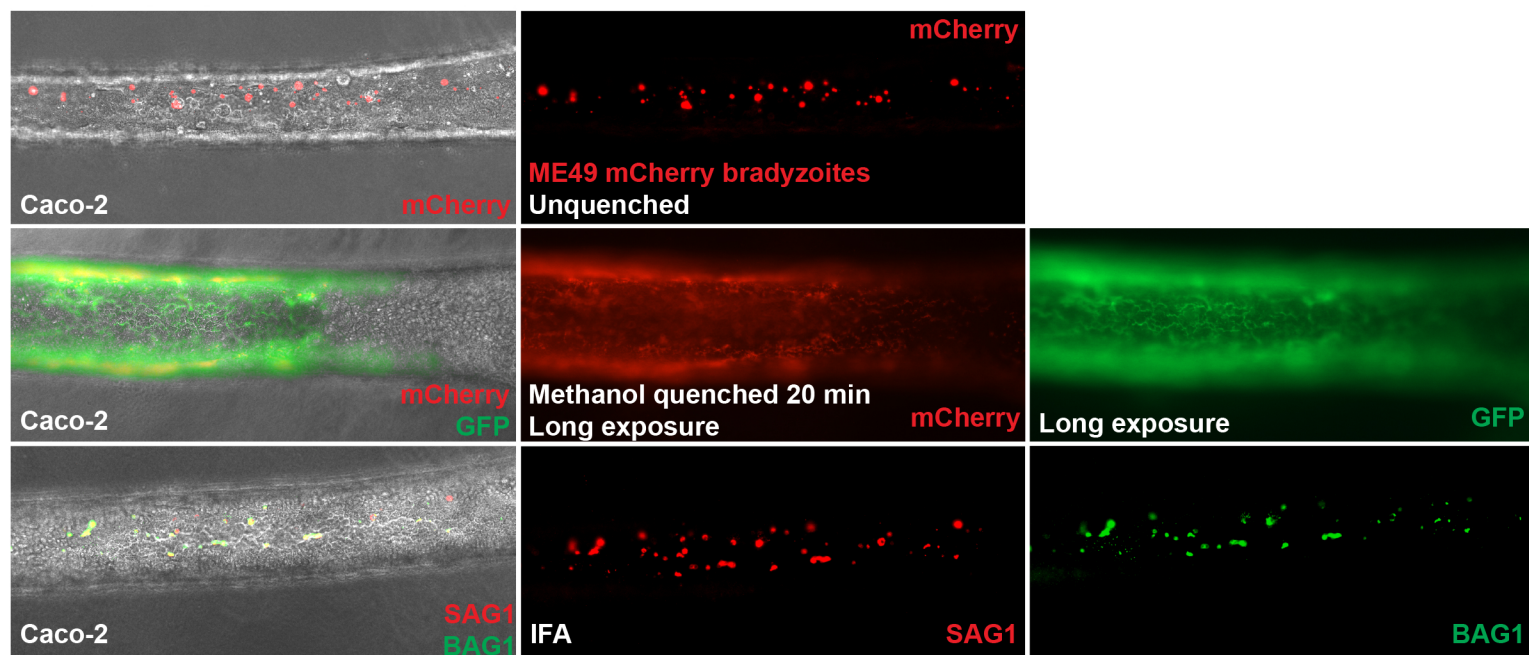

Supplement: S11 Fig — (A) Show a lumen infected with ME49 mCherry brain bradyzoites after 48 hours post-infection. Top lumen shows unquenched parasites. Bottom lumen shows the same lumen with the mCherry quenched after incubation with methanol for 20 min. Bottom lumen was over exposed to detect any remnant of mCherry in the lumen. (B) Show a lumen infected with ME49 mCherry brain bradyzoites after 72 hours post-infection. Top lumen shows unquenched parasites. Middle lumen shows the same lumen with the mCherry quenched after incubation with methanol for 20 min. Bottom lumen shows an IFA against SAG1 and BAG1. Middle and bottom lumen were over exposed to detect any remnant of mCherry or GFP in the lumen. (PDF) [file pntd.0012855.s011.pdf]

# Explants of villi after 24 h

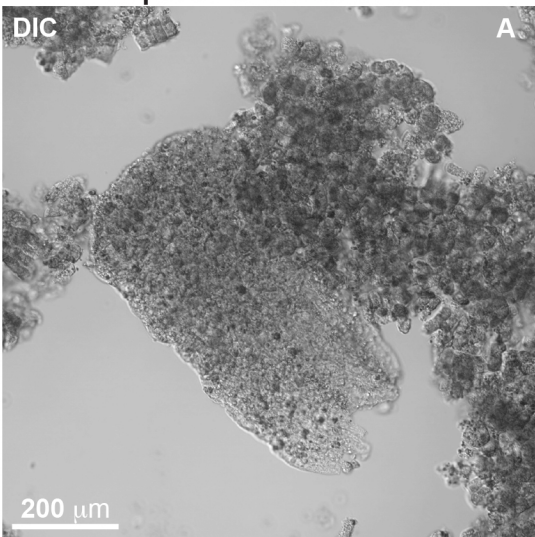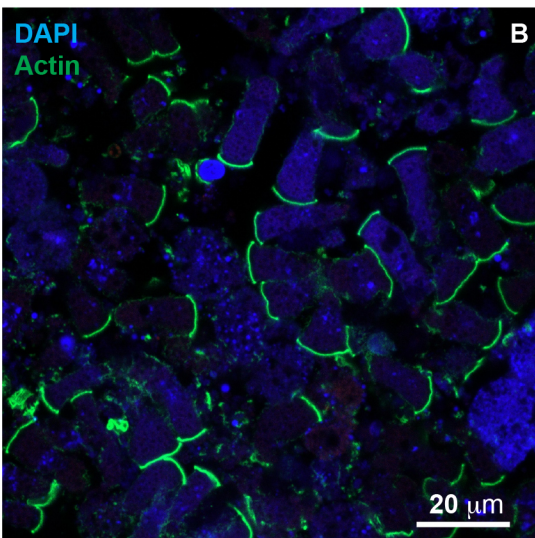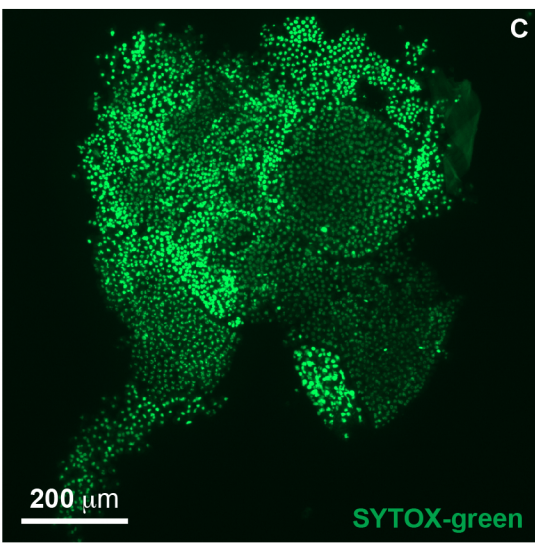

Supplement: S12 Fig — (A) Intact villus surrounded by villi material. (B) Free floating enterocytes due to the disintegration of villi during incubation. (C) Cellular viability assessed using SYTOX Green, with dead cells highlighted by green-stained nuclei. (PDF) [file pntd.0012855.s012.pdf]
