# Supplementary material for: Modeling Toxoplasma gondii-gut early interactions using a human microphysiological system
Source: PLoS Negl Trop Dis. 2025 Feb 4;19(2):e0012855. doi: 10.1371/journal.pntd.0012855 (PMC12136440; doi:10.1371/journal.pntd.0012855)
Supplement: S3 Fig — (A) Representative epifluorescent images of jejunum at 3 days post-infection showing SAG1-positive and BAG-negative parasites in the stroma. (B) Representative epifluorescent images of jejunum at 5 days post-infection showing SAG1-positive and BAG-negative parasites in the stroma. Intestines are stained for WGA (green), SAG1 (red), BAG1 (red), and nuclei (blue, DAPI). (PDF) [file pntd.0012855.s003.pdf]

A

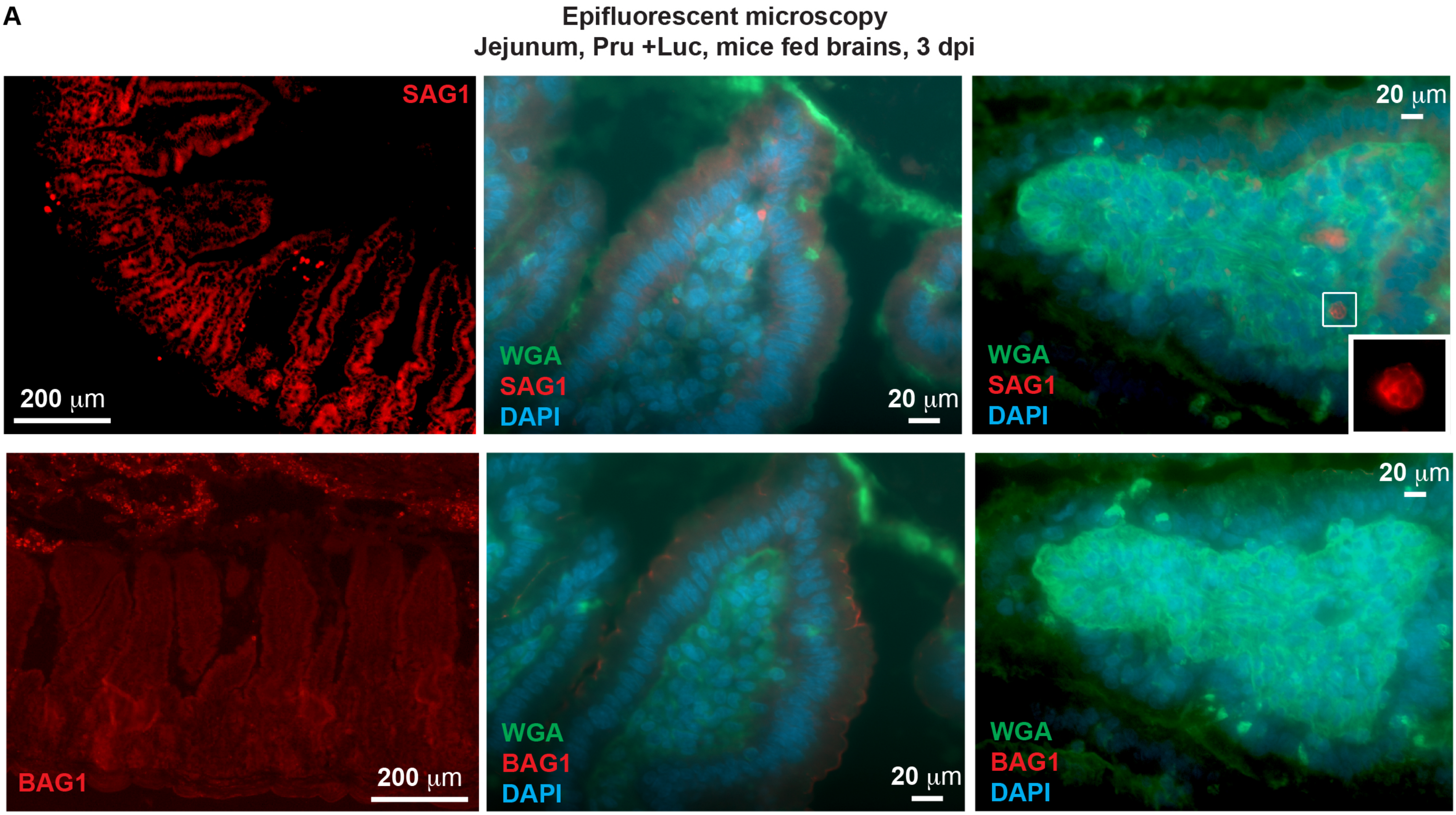
Epifluorescent microscopy
Jejunum, Pru +Luc, mice fed brains, 3 dpi
SAG1
200 μm
WGA
SAG1
DAPI
20 μm
20 μm
WGA
SAG1
DAPI
BAG1
200 μm
WGA
BAG1
DAPI
20 μm
20 μm
WGA
BAG1
DAPI

B

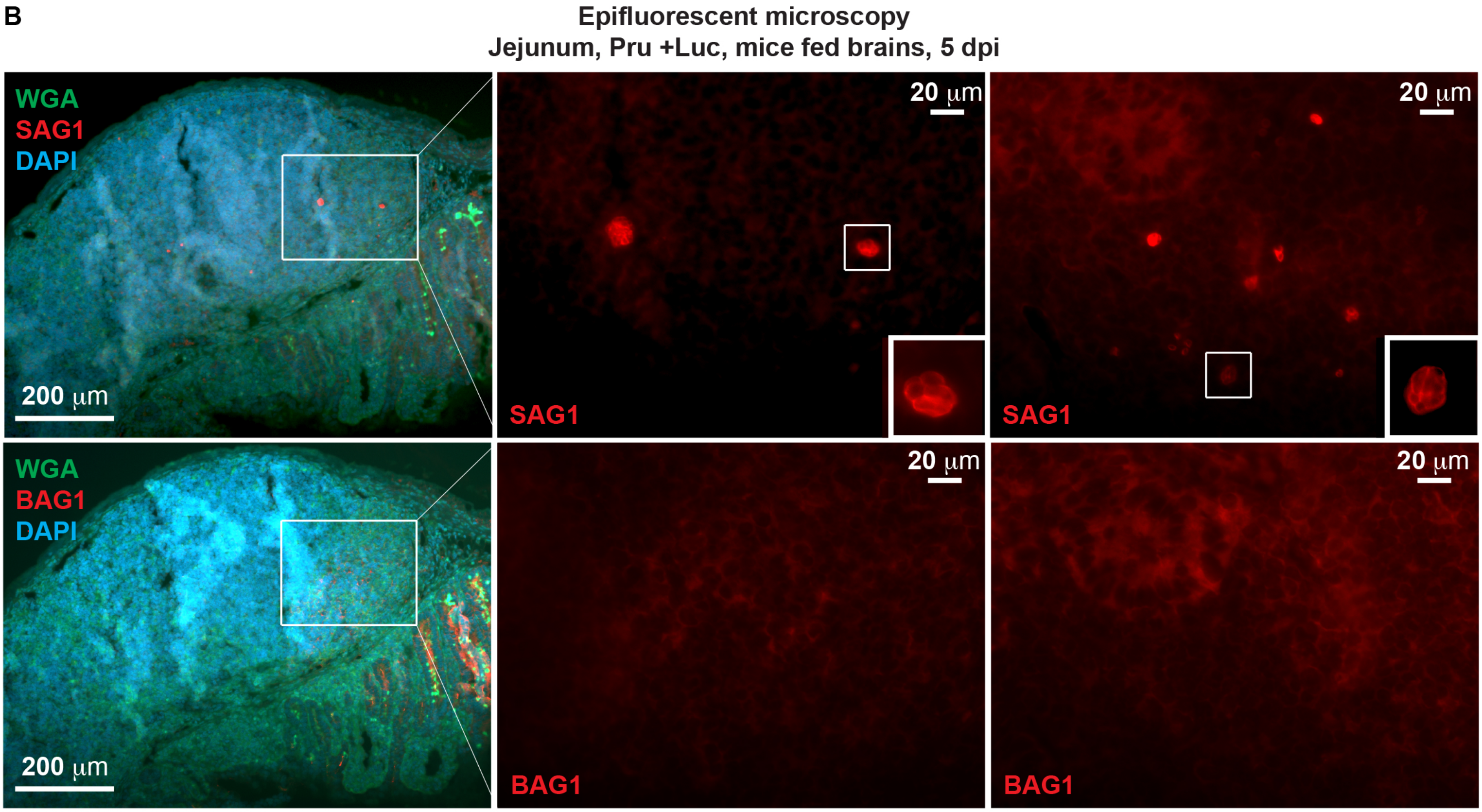
Epifluorescent microscopy
Jejunum, Pru +Luc, mice fed brains, 5 dpi
WGA
SAG1
DAPI
200 μm
20 μm
SAG1
20 μm
SAG1
WGA
BAG1
DAPI
200 μm
20 μm
BAG1
20 μm
BAG1
