# Supplementary material for: Modeling Toxoplasma gondii-gut early interactions using a human microphysiological system
Source: PLoS Negl Trop Dis. 2025 Feb 4;19(2):e0012855. doi: 10.1371/journal.pntd.0012855 (PMC12136440; doi:10.1371/journal.pntd.0012855)
Supplement: S4 Fig — (A) Representative epifluorescent images of jejunum showing SAG1-positive parasites in the stroma and close to the muscular layer. Bottom insets in (A) show a possible immune cell infected by T. gondii. (B) Representative confocal images of jejunum showing parasites replicating in the stroma. Parasites were stained using polyclonal antibodies against T. gondii. (C) Representative confocal 3D projection of jejunum showing SAG-1 positive parasites replicating in the stroma. Intestines are stained for WGA (green), SAG1 (red), polyclonal antibodies against T. gondii (green), and nuclei (blue, DAPI). (PDF) [file pntd.0012855.s004.pdf]

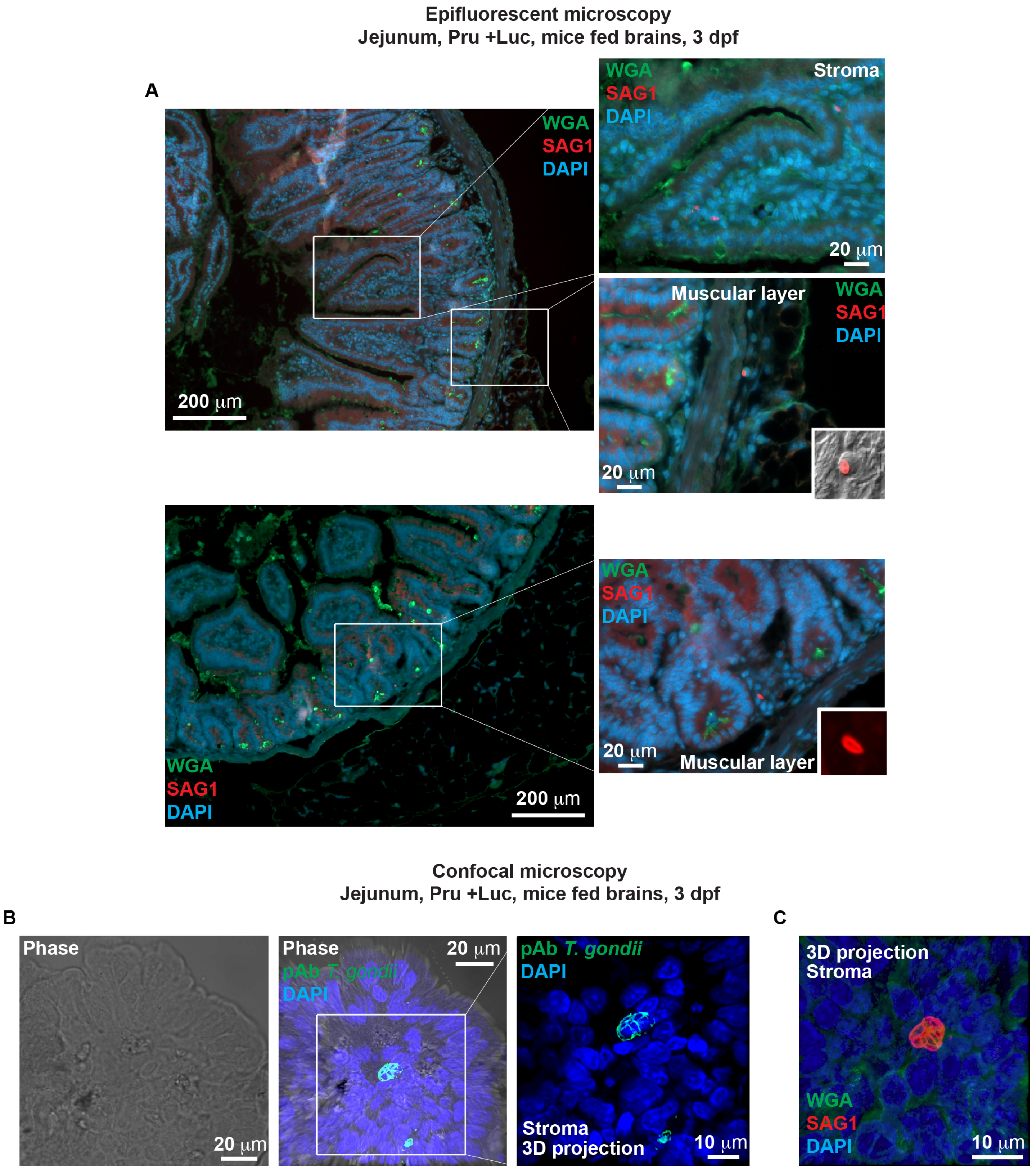

Epifluorescent microscopy
Jejunum, Pru +Luc, mice fed brains, 3 dpf
A
WGA
SAG1
DAPI
200 μm
Stroma
20 μm
Muscular layer
20 μm
Muscular layer
Confocal microscopy
Jejunum, Pru +Luc, mice fed brains, 3 dpf
B
Phase
20 μm
Phase
pAb T. gondii
DAPI
10 μm
Stroma
3D projection
C
3D projection
Stroma
