# Supplementary material for: Modeling Toxoplasma gondii-gut early interactions using a human microphysiological system
Source: PLoS Negl Trop Dis. 2025 Feb 4;19(2):e0012855. doi: 10.1371/journal.pntd.0012855 (PMC12136440; doi:10.1371/journal.pntd.0012855)
Supplement: S5 Fig — (A) Confocal images of female and male jejunum showing T. gondii parasites replicating near areas with excessive cell infiltration. (B) Confocal image of tachyzoites replicating surrounded by CD45-positive cells. (C) 3D projection of tachyzoites replicating within the stroma. (D) Epifluorescent image of tachyzoites (red) near the endothelium. RBC states for red blood cells. (E) Representative epifluorescent images of uninfected jejunum stained against CD45. (F) and (G) Show CD31-positive cells, as a result of our best attempt to obtain CD31 in the jejunal villi after 3 days post-feeding. Intestines are stained for polyclonal antibodies against T. gondii (green), CD31 (red), CD45 (green), and nuclei (blue, DAPI). (PDF) [file pntd.0012855.s005.pdf]

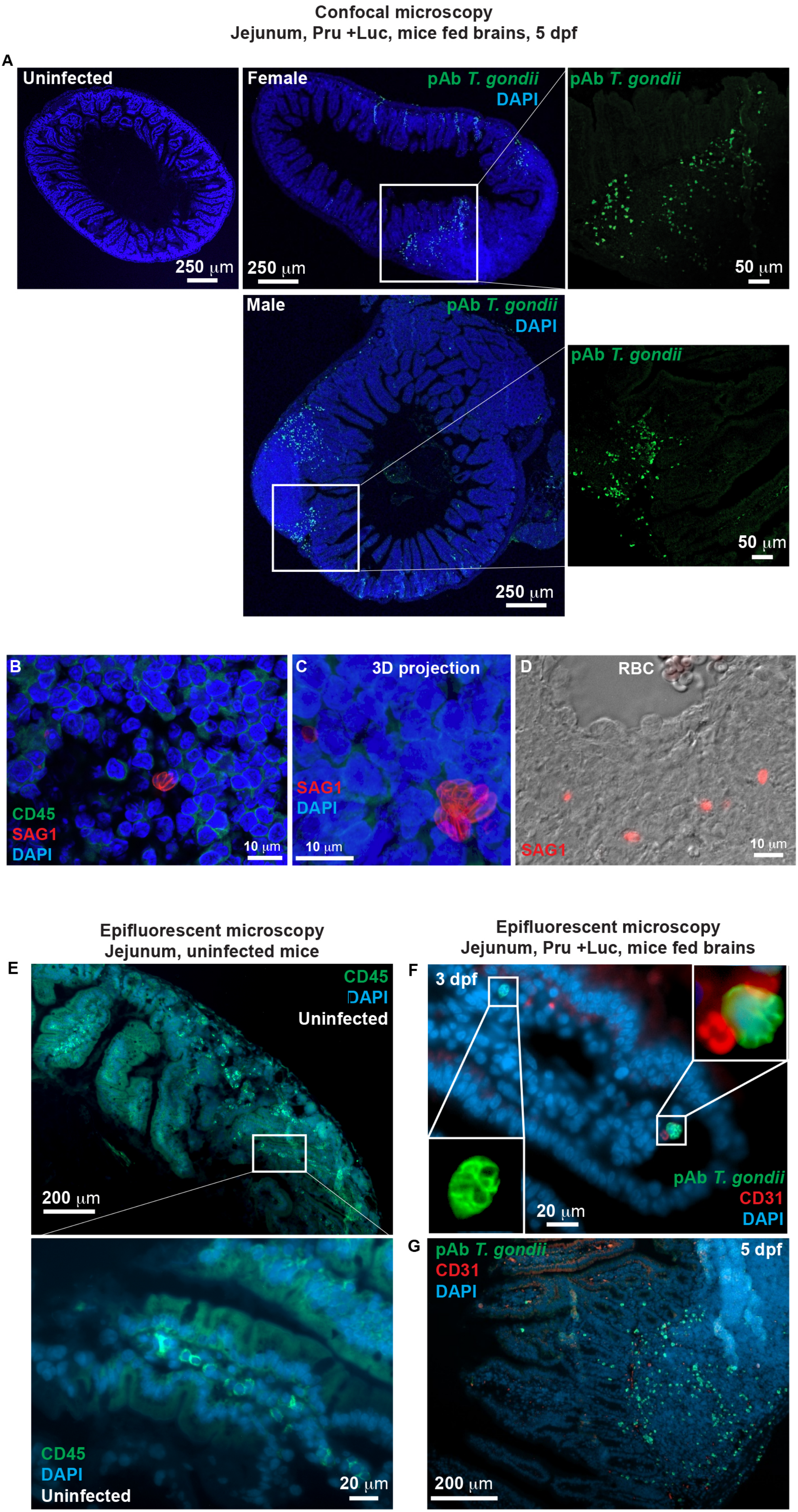
Confocal microscopy
Jejunum, Pru +Luc, mice fed brains, 5 dpf
A
Uninfected
250 μm
Female
pAb T. gondii
DAPI
250 μm
pAb T. gondii
50 μm
Male
pAb T. gondii
DAPI
250 μm
pAb T. gondii
50 μm
B
CD45
SAG1
DAPI
10 μm
C
3D projection
SAG1
DAPI
10 μm
D
RBC
SAG1
10 μm
Epifluorescent microscopy
Jejunum, uninfected mice
E
CD45
DAPI
Uninfected
200 μm
CD45
DAPI
Uninfected
20 μm
Epifluorescent microscopy
Jejunum, Pru +Luc, mice fed brains
F
3 dpf
pAb T. gondii
CD31
DAPI
20 μm
G
pAb T. gondii
CD31
DAPI
5 dpf
200 μm
