# Supplementary material for: Modeling Toxoplasma gondii-gut early interactions using a human microphysiological system
Source: PLoS Negl Trop Dis. 2025 Feb 4;19(2):e0012855. doi: 10.1371/journal.pntd.0012855 (PMC12136440; doi:10.1371/journal.pntd.0012855)
Supplement: Attachment — Submitted filename: Response to Reviewers.docx [file pntd.0012855.s020.docx]

Dear *PLoS Neglected Tropical Diseases* editors,

Thank you for this opportunity to significantly improve our manuscript. The reviewers have made some great suggestions, and we are happy that they took the time for such a careful review. We have addressed each of their critiques on a point by point basis (our response is in blue below). Thanks again, and we hope that this manuscript is now suitable for publication in *PLoS Neglected Tropical Diseases*.

Best Regards,

Laura Knoll

**Methods**

Reviewer #1: In general the experimental methods are quite clear, however there are many occurrences where method specific details are lacking. For example number of samples, number of mice, ratio of positive to negative samples, appropriate controls.
How the mice were infected with a consistent number of cysts to ensure robust, consistent infections that can be compared between samples and sexes when the cysts are brain derived is missing from the manuscript and is vital information to draw conclusions comparing strains, sexes and distributions. This information is clear for in vitro derived cyst infections.

We thank the reviewer for their helpful suggestions. We apologize for the insufficient information previously provided. In response, we have included the number of mice per experiment and the total number of mice in both the Methods section and the figure legend. For example, figure legend 1 now includes information about the number of events captured, the number of mice analyzed, and the number of cysts used for infections. We have also clarified the details regarding the corresponding controls.

Some of the examples of new information are:

We added in methods regarding mice infections: ***“****Briefly, two mice were fasted overnight (70) and consumed the brains containing in vitro cysts within a 1-hour window. Each mouse was fed with two brains, each containing ~2 x 10^2^ cysts per brain. The number of cysts were determined isolating brains from two infected mice from the same population, as above described”.*

other example: *“To study T. gondii intestinal interactions followed by the natural route of infection, three males and four females Swiss Webster mice were infected by consuming brains containing cysts (refer to figure legends to see the mice number in each time point). Each mouse ingested two brains with a total approximated dose of 4x10^2^-5x10^2^ brain cysts. The number of brain cysts were determined by isolating brains from two infected mice from the same population, as above described”.*

And in the legend of figure 1 now it reads: *“Images represent single events captured in independent infected mice after 3 days post-ingestion. Single mouse ingested brains containing ~4 x 10^2^ cysts”.*

Reviewer #2: This is a very useful paper that provides interesting information and new data about bradyzoites, differentiation in vitro and the mechanism of bradyzoite transport across epithelial borders in the intestine. The statistics and sample sizes are adequate for the data presented. All regulatory requirements have been met.

A minor correction in the introduction line 67 is that Toxovax is a licensed attenuated strain (S48 strain) veterinary vaccine for sheep for T. gondii.

We thank Reviewer #2 for this correction. We have added the information to improve the introduction and its corresponding reference. Now, it reads: ***“****Despite high rates of infection, there are currently no vaccines available for humans, and treatment options are limited. A live attenuated vaccine called ToxoVax (MSD, New Zealand) effectively reduces losses in breeding sheep, highlighting the potential for future human vaccines (9)”.*

**Results**

Reviewer #1: I have broken my review into what I consider the three big results sections of the manuscript:
1. in vivo interactions in the gut:
The first 6 figures of this manuscript do not fit well with the title and appear to be included to justify the use of MPS to better probe cyst gut interactions. I would suggest that these be included as a single main text figure explaining the challenges of in vivo systems with supporting figures in the supplementary data if necessary. Including it ‘as is’ detracts from the exciting data presented in the later figures using MPS, that more closely fit the paper title.

We agree with Reviewer #1 that a redistribution of the figures would enhance the clarity and understanding of our results. We combined figures 1, 2, and 3 into a new Figure 1. This figure retains key new observational results that we believe will be of interest to the field. Similarly, Figures 4, 5, and 6 have been merged into Figure 2, and the remaining information has been moved to the supplemental material.

If the data are to remain in their current format the text needs to be clarified to state; the numbers of observations and samples analyzed, and the following specific issues need to be addressed: The data presented in figures 1 – 3 of this manuscript provide new observational information about parasite infection of the gut in the first 3 – 5 days post infection. It should be clarified that this is a limited number of observations that cannot be quantified, and the number of animals, individual samples (e.g. number of villi analyzed, number of foci imaged etc.) and specific methodologies need to be included.

Excellent suggestion, we quantified the number of events observed in the intestines, specifically in terms of villi and bumps infection. We also clarified in the text that certain events, such as the detection of parasites in scraped villi, are limited and cannot be accurately quantified. Each of these points has been addressed in the result section and their corresponding figure legends.

Some examples of the modifications in results section include:

*“We observed infrequent foci of infection after examining hundreds of scraped villi from each infected mouse (Fig. S1). We could not quantify the findings, given that the number of observed events were limited”.*

In figure legend 1:

*“Data in* ***(J)*** *represents a combination of at least 12 independent observations in different sections of the same or different infected mouse. Data in* ***(L)*** *represents observations of bumps in infected sections of the same or different mouse”.*

For Figure 1. I cannot discern how many mice had their villi scraped, how many villi were analyzed to detect parasites and I’m unclear if the 6 examples shown are the only parasites found or if these are representative images of parasites in those specific cells and quantification could be included. Are heavily infected villi missing from the analysis because they are more fragile and therefore lost? Or do the authors believe this low density of infection is the natural occurrence? How many cysts were used to establish the infection? Could optimizing the number of cysts or the preparation of cysts increase the number of parasites per villi or the number of usable villi that can be obtained.

We agreed that methods clarification was needed, so we expanded the information in the results and the methods sections. We added the number of mice we used for infecting to obtain the infected brains, as well as the number of mice that were infected by ingesting the infected brains. We also added the approximate number of cysts that each mice ingested according to our cysts counts for the M4 strain in Swiss Webster mice.

The methods now read: *“Subsequently, 1 x 10^4^ tachyzoites of the M4 strain were infected in mice to develop chronic infection. At least four brains containing cysts were collected and served as food for two healthy mice, following the natural route of infection. Briefly, two mice were fasted overnight (70) and consumed the brains containing in vitro cysts within a 1-hour window. Each mouse was fed with two brains, each containing ~2 x 10^2^ cysts per brain. The number of cysts were determined isolating brains from two infected mice from the same population, as above described”.*

We clarified the methodology for scraping the jejunum to release the villi, and we mention the low efficiency for detecting infected areas between hundreds of uninfected villi. In the section “Bioluminescence imaging reveals dynamic distribution of *T. gondii* Infection in mice intestines”, we state that we faced challenges in detecting parasites and infected cells in the scraped intestines. In response to reviewer comment, we think the infrequent foci of infection could be due to the loss of villi directionality, villi/crypt integrity, and possible stroma damage due to the mechanical force when scraping.

The text now says: *“We infected a total of six mice across three independent experiments, each mouse fed two brains containing approximately 4 x 10² brain cysts of the M4 strain. To detect the parasites in the jejunal villi, we sacked the mice after 3 days post-feeding and scrapped the jejunal villi”…. “Although we collected numerous scraped villi from the jejunum of a total of six infected mice, detecting extracellular parasites or infected cells was challenging”…. “We observed infrequent foci of infection after examining hundreds of scraped villi from each infected mouse (Fig. S1). We could not quantify the findings, given that the limited number of observed events were limited”.*

For Figure 2 and supplementary Figure 1. IVIS images from 1 uninfected and 2 male and 2 female mice are shown for each time point and more intestines that have been isolated. However, the numbers that these are representatives for is not included. Likewise for the number of mice for quantifications in D and E, for each of the 2 experiments how many male and female mice were analyzed, and were all foci identified used for flux quantifications? To draw broad conclusions about distribution throughout the gut and the number of foci, several mice (5+ of each sex, in my opinion) would have been analyzed and the infection dose would have to be consistent, or the differences could be due to infection dose rather than mouse sex as implied in the text. More detail is required for these experiments.

We agree and apologize for the confusion. We have now included the number of mice used in the IVIS data analysis. We also added the approximate number of brain cysts ingested per mice.

Some of the modifications in the text include:

In methods: *“To study T. gondii intestinal interactions followed by the natural route of infection, three males and four females Swiss Webster mice were infected by consuming brains containing cysts (refer to figure legends to see the mice number in each time point). Each mouse ingested two brains with a total approximated dose of 4x10^2^-5x10^2^ brain cysts. The number of brain cysts were determined by isolating brains from two infected mice from the same population, as above described”.*

In results: *“We therefore infected mice with PruΔHPT:luciferase strain to obtain brain cysts. After day 28 post-infection, we isolated the brains and fed mice with brain containing cysts (Fig. 1Ci). Each mouse ingested an approximate dose of 4x10^2^-5x10^2^ brain cysts”.*

We recognize the importance of including an adequate sample size when reporting sex differences. Therefore, we have removed any speculative statements regarding sex differences from the text to prevent overstating potential variations in infection. However, we have retained the information related to sex in the Methods section to ensure full transparency. The primary objective of the IVIS experiments was to identify foci of infection for subsequent tissue analysis.

The graphs in panels D and E, which previously showed male and female data separately, have been replaced with graphs showing combined data from both sexes. All foci of infection were analyzed using Flux. These graphs represent data from a total of seven mice—three mice at 3 days post-infection and four mice at 5 days post-infection. This information has been updated in the text.

Figure 3 and Supplementary figures 2 – 4. Like with figure 1 this is interesting data that shows tachyzoites are the only stage present in the gut at 3- and 5-days post infection and that these parasites can be found in different locations and might cause changes to the gut (S4). Also, like Figure 1 quantification of the frequency of these observations is missing.

As mentioned above, we have redesigned Figure 1. The new Figure 1 now includes the data previously presented in Figure 3. We decided to retain only representative images of infected jejunum at 5 days post-infection, while the additional data is available in the supplementary material. We also included information regarding the frequency of these observations.

Additionally, how the infections were controlled to be comparable between male and female mice, between time points and replicates is not included in the legend or methods.

As previously mentioned, in response to the reviewer’s comments we removed the interpretation regarding sex differences from the manuscript.

The bumps associated with foci of parasite replication are also interesting, but quantification of how often a bump is found and the number of slices that were analyzed to determine they are not found in uninfected mice and that they are only found at “replication/infection hotspots” is needed.
The low number of observations in these experiments is a clear justification for why the MPS is such a good way to look at the initial interactions but it is my opinion that the narrative would benefit from condensing and clarifying the message of these data.

We thank Reviewer #1 for their valuable feedback. The bumps in the intestines were clearly visible in every section of the infected areas. The frequency of the bumps and associated infection data were quantified and are now included in the results. The methods section was also modified to include the number of slices analyzed.

Now, in methods, it reads:

*“Paraffin-embedded intestines were serial cut in at least 4 sections per slice to facilitate antibody comparisons. Infected sections of the intestine were processed at least three times for detecting parasites in different planes. Giving a total of at least 36 sections of foci of infection per conditions”.*

In figure legend:

*“Data in* ***(J)*** *represents a combination of at least 12 independent observations in different sections of the same or different infected mouse”.*

The bioluminescent regions observed via IVIS correlated well with the visual detection of bumps by the naked eye. We added an image of an enlarged infected intestine, captured during the IVIS imaging process. These results will be of interest to the field as they provide a potential method for studying intestinal infections in laboratories without access to fluorescent or bioluminescent strains, or IVIS equipment.

In figure legend:

*“B’ inset in top figure shows a bumped area of the intestine that corresponds with the bioluminescent region detected by IVIS”.*

In results:

*“The enlarged areas were detected by naked eye, colocalized with bioluminescent areas seen by IVIS and present in all the intestinal sections analyzed at 5 days post-ingestion. Hundred percent of the bumps were infected or surrounded by parasites”.*

2. Optimization of an in vitro differentiation assay.
The optimization of the in vitro differentiation assay to yield such high differentiation rates is going to be really useful to the field and the data seem robust. As with the previous set of comments I also believe there are too many examples and not enough quantification. And would suggest including one representative image of how the quantification was done is sufficient for each staining method (BAG1 or DBA) or fluorescent line (EGS) accompanying the quantification making a single figure. The images from different time points and different lines with the same methodology are overwhelming and doesn’t add much.

We agree with Reviewer #1 and simplified this section. We included representative images of the staining used for quantifying the switching process of the parasites. This data is now presented in Figure 2.

(minor) Could it be made clear for each condition not only the temperature and medium the parasites are grown in but also the CO2 level.

Our in vitro encystment is performed in a thermostatic incubator 33 °C with no regulation of gases. We tightly closed the flasks to avoid gas exchange during the time the parasites were incubated at 33 °C. We added this information to the methods and results.

We also used RPMI 1640 without sodium bicarbonate, so we updated the information in methods. It now reads: *“Briefly, HFF cells were infected with 5 x 10^5^ – 2 x 10^6^ tachyzoites for 2 h. Then, extracellular parasites were removed and differentiation media (RPMI 1640 without sodium bicarbonate (Sigma-Aldrich), 1% FBS, and 40 mM HEPES, at pH 8.1.) was added. Infected cells in differentiation media were incubated at 33°C for up to 12 days in a thermostatic incubator under normoxic conditions with ambient CO_2_ concentrations”.*

In results: *“The new differentiation condition includes culturing tachyzoites in media with a pH 8.1 at 33°C (39), in a thermostatic incubator under normoxic conditions with ambient CO_2_ concentrations”.*

(minor) Line 258/9 what does this statement mean?

We wanted to summarize molecular and physiological features that in vitro bradyzoites share with brain cysts, such as: positive for DBA and BAG1, negative for SAG1, rounded shape structures, slow replication parasites, and resistance to pepsin digestion. We added this clarification in the text, now it reads: “*In vitro* bradyzoites differentiated at 33°C in switching media exhibited molecular and physiological features similar to brain cysts, such as: positive for DBA and BAG1, negative for SAG1, rounded shape structures, slow replication parasites, and resistance to pepsin digestion”.

Figure 6. A) ME49 mCherry constitutively expressed mCherry, how do you discern they are bradyzoites replicating in the HFF and Caco-2 cells?

We thank reviewer #1 for pointing out this inconsistency about Figure 6A, this was a typo. In agreement with the previous reviewer’s comments, we incorporated the content of Figure 6 in the Supplemental Figure 2F. We changed this statement from Figure 6A. Now, the new panel reads: Replication of ME49 mCherry parasites after 72 hpi as bradyzoites.

(Exp) C/D If I am reading the legend correctly and only 3 (feeding, EGS) or 2 (gavage, ME49) mice were infected with each dose for each strain. This is not enough mice to draw conclusions about virulence. The two infection methods also cannot be compared as previous work has shown infection route changes dissemination and virulence. Furthermore, there is not enough context for these infections, how many in vivo derived cysts are required for mice to succumb to infection with each of these lines? To make the statement (figure title) that “in vitro generated bradyzoites exhibit virulence similar to that of brain cysts” this is required. The text also suggests ME49 was also fed to mice and a mouse (out of an unknown number) did become chronically infected with a dose of 1x104 cysts.

We agree that oral gavage and natural ingestion of cysts cannot be directly compared with those of a few mice. Our point was to show that the in vitro bradyzoites cysts were orally infectious by natural ingestion, so we have removed the data from the oral gavage experiment. As per the reviewer’s suggestion, we have added an additional experiment in which 3 mice were naturally infected with 7 x 10^4^ cysts. While these mice did not succumb to the infection, they developed brain cysts, and this information is now included in the results and methodology sections.

To further characterize the in vitro-generated bradyzoites, we performed an infection experiment where we introduced in vitro cysts into a piece of bread for oral administration, ranging different numbers of in vitro cysts of the EGS strain. This experiment demonstrates that in vitro-derived bradyzoites of the EGS strain are infectious to mice. Our data show that 1 x 10^5^ cysts are required for mice to succumb to the infection. These results are shown now in Fig. 2G. Unfortunately, we could not generate in vivo derived cysts of the EGS given their high virulence so we agree with the reviewer that we cannot say “in vitro generated bradyzoites exhibit virulence similar to that of brain cysts”. We removed that statement.

The methods now say: *“Three male Swiss Webster mice per dose were naturally infected with in vitro cysts of EGS strain: doses were 1 x 10^2^, 1 x 10^3^, 1 x 10^4^, or 1 x 10^5^. For ME49 in vitro cysts, natural infection doses were 1 x 10^4^ (one mouse) or 7 x 10^4^ (three mice)”.*

The results now say: *“One mouse fed ~1 x 10^4^ and three mice fed ~7 x 10^4^ ME49 mCherry in vitro-differentiated cysts developed chronic infection after 28 days, as evidenced by cyst isolated from the brain. In contrast, mice fed ~1 x 10^4^ EGS in vitro cysts did not exhibit brain cysts formation or succumbed to the infection after 28 days post-feeding (Fig 2G). To evaluate the virulence of EGS in vitro cysts further, three mice per group were fed with EGS in vitro cysts ranging from ~1 x 10^2^ to ~1 x 10^5^. Only mice fed with ~1 x 10^5^ in vitro cysts succumbed to the infection after 11-13 days (Fig. 2G6C). Brain cysts were not detected in the remaining EGS-infected mice, likely due to the nature of this type I/III hybrid strain.*

Supplementary Figure 6. This section of the text is confusing and I’m not sure what the goal was or the conclusion. The opening sentence seems quite misleading.

We agreed that these results were out of context, so we have removed Supplementary Figure 6 and its corresponding text.

3. Modeling parasite-gut interactions with MPS.
To me these are the most important and exciting data presented in the manuscript and should be the major focus based on the title. I think the formatting of these sections is much clearer than the previous ones and the only overall request is further details on how experiments were standardized to allow comparisons. More specific comments:
The MPS acronym is used widely without explanation of what it stands for/is outside of the title, which does not include the (MPS). I would like a little expansion of the system lines 288-295.

We thank reviewer #1 for their helpful comments and for the interest in our results. We changed the title in agreement with their comment. Now, the new title reads: “*Modeling Toxoplasma gondii-gut early interactions using a human microphysiological system”*

The acronym 'MPS' was first introduced in the introduction, where we defined it as 'microphysiological system.' We also added the acronym meaning MPS again at the beginning of the results section and in the figure legends. We expanded the explanation of the controls used in our previous report and the controls used in this report. Now, it reads: *“Our group has successfully used the microphysiological systems (MPS) and Caco-2 cells to model T. gondii tachyzoite-specific interactions during the infection and study the innate immune response (35). We turned to these devices to model early intestinal bradyzoite infection using Caco-2. MPS previously demonstrated confluent distribution throughout the lumen and along with the presence of mucus-producing goblet cells marker MUC2A, the tight junction protein ZO-1 (confirming the stability of the cells forming the lumen), and the microvilli marker villin (35). We confirmed the homogeneous and close distribution of Caco-2 cells in the current study by actin staining. Barrier functionality and luminal coverage were validated by the maintenance of FITC-dextran within the lumen. We tested the viability of Caco-2 cells in the MPS by infecting them with RH mCherry tachyzoites and detecting active replication after 48 hours post-infection”.*

298. How do you ensure the me49 mCherry line is a pure bradyzoite line, as far as I am aware, mCherry expression is constitutive in this line and it is only the EDS that has stage specific expression of fluorescent proteins.

We thank the reviewer for this valuable observation. After characterizing in vitro encystment, we can confirm that we achieved >96% switching under the new conditions reported herein, which increases the chances of the cells becoming bradyzoites. In addition to the high efficiency of generating in vitro bradyzoites, we can also confirm that the in vitro cysts were digested and activated using pepsin, further increasing the likelihood of selecting for viable bradyzoites.

Figure 7B quantification of mCherry area after seeding MPS with acid pepsin digested bradyzoites shows low fluorescence at days 4—6 and really the signal isn’t robustly detected of different until day 8 post infection, Supplementary 7 shows images at 5 and 6 dpi, based on the quantification in the main figure I would not have expected to see parasite growth, so the inclusion seems odd. The label I believe should read dpi and says 5hpi.

We thank the reviewer for this observation. Figure 6 (now figure 3) and Supplementary Figure 7 (now Fig. S8) present independent experiments and are not complementary. Supplementary Figure 7 (now Fig. S8) shows our in vitro ME49 bradyzoites after just 2 minutes of pepsin digestion. As we explained in results, after just 2 min of pepsin digestion, we did not observe parasite replication in Caco-2 lumens. A similar lack of replication was observed with EGS parasites after 2 min of pepsin, as shown in Supplementary Figure 8 (now Fig. S9).

After this low bradyzoite infection rate, we decided to extend the pepsin treatment and investigate its potential as an activator of bradyzoites. To evaluate this, we returned to 2D culture systems. In the previous Figure 7B, we show the effect of pepsin in activating bradyzoites up to 2 hours of treatment during their invasion of HFF cells, while Figure 7C compares the infection of three different cell lines with bradyzoites that had been digested for 30 minutes with pepsin—the optimal condition (now Figure 3B, C). Following this, we returned to the devices to test the new conditions for pepsin digestion.

To avoid confusion, we changed the heading to: *“Optimization of human cell lines for use in microphysiological systems”.* We also added to the results: *“We tested the role of pepsin in bradyzoite activation in 2D monolayers. We incubated ME49 mCherry brain cysts with pepsin from 2 min up to 2 h, and then monitored parasite replication over 9 days in HFF monolayers”.*

And

*“Given that our MPS are seeded with Caco-2 cells, we ensured that bradyzoites infect Caco-2 cells under our new activation conditions. We compared 30 min pepsin-activated brain bradyzoite infection in Caco-2 vs HFF monolayers”.*

Additionally, we have corrected the typo in Supplementary Figure 7A, now Supplementary Figure 8A. Thank you for catching that.

(Exp) Figure 8A these experiments use ME49 mCherry parasites then also use SAG1 with alexa 594 to “stage” parasites. If mCherry signal is completely lost with the authors fixing method, then this could be acceptable, but no controls are provided to show this. The resolution is not high enough to determine if the signal is peripheral (as would be expected for SAG1) or cytosolic (as would be expected for mCherry). The co-labelling could just be BAG1 and residual mCherry, the SAG1 with Alexa 594 secondary needs more controls to be robust.

We apologize that we did not include the controls that we performed. We had controlled for the loss of mCherry fluorescence by using methanol in our experiments. We have included a new Supplementary Figure that demonstrates the control of parasite replication in lumens at 48- and 72-hours post-infection. Both panels show the absence of mCherry fluorescence following treatment with methanol. The procedure for quenching mCherry fluorescence is described in detail in the Methods section.

In the methods it reads: *“DBA, SAG1, or BAG1 positive vacuoles, infected monolayers were fixed with 4% paraformaldehyde for 30 min or ice-cold methanol for 20 min. Methanol was also used to quench mCherry or GFP fluorescence in parasites”.*

In the results, it now reads: *“At 3 days post-infection, parasites were fixed, mCherry fluorescence was quenched using methanol, and then parasites were stained with SAG1 and BAG1 antibodies”.*

8B The resolution of the images shown is difficult to be sure that there is no remaining GFP signal from bradyzoite, especially with the auto fluorescence seen in the MPS. The dynamics presented in this figure are interesting and the technology provides a unique way to look at this that has been inaccessible in vivo. However, there is no quantification and the kinetics and dynamics seen here in one CACO-2 MPS needs to be repeated and quantified to present a stronger finding.

We thank the reviewer for this valuable suggestion. We agree that further characterization and quantification were necessary. To address this, we infected Caco-2 lumens with our EGS strain and quantified tachyzoite conversion by counting red parasitophorous vacuoles (tachyzoites expressing mCherry and SAG1). We quantified these events in 36 different Caco-2 lumens of the seven plates presented in the graph. The updated results are presented in a new figure 4B, showing the average values of the quantifications.

The results now read: *“To monitor stage conversion in real-time, we infected Caco-2 lumens with in vitro bradyzoites of the EGS strain and quantified tachyzoite conversion by counting red PVs (mCherry/SAG1 expressing parasites). At 2 day post-infection, >90% of PVs contained GFP-expressing bradyzoites (Fig. 4B). Consistent with the ME49 strain, tachyzoite conversion was clearly detected by day 3 post-infection in 30% of vacuoles (Fig. 4B). By day 4 post-infection, tachyzoites were present in 70% of PVs, and near complete tachyzoite conversion was evident by 5 days post-infection with only mCherry-expressing EGS parasites detected (Fig. 4B)”.*

The methods now read: *“For the quantification of tachyzoite conversion, seven plates were infected with 3 x 10³ in vitro bradyzoites. EGS tachyzoites were switched to the conditions described above and maintained for at least 7 days. Bradyzoite conversion to tachyzoites was monitored in the device using the EVOS FL Auto system and assessed by detecting PVs positive to mCherry/SAG1. Results are shown as a percentage of the average number of red vacuoles in each lumen per plate, 36 lumens total were quantified”.*

Figure 9 reads like an extension of Figure 1 and doesn’t flow from the previous MPS results. It is also observational, like Figure 1.

We apologize that we did not clearly tie this figure to the MPS results. We believe this figure is necessary as it shows that the events occurring in the MPS also occur in the infected intestine. We agree that this figure may not be ideally placed, as it could disrupt the flow of the section on the devices; however, we believe these results are complementary. Rapid transmigration was observed in MPS and in the intact intestine, highlighting that this event is not an artifact of the MPS.These experiments will serve as a foundation for future studies aimed at these early interactions.

We clarified the results to say: “During the early stages of bradyzoite infection in the MPS, we observed parasites localizing to the collagen I/fibronectin matrix within 20 hours post-infection. This finding suggests that bradyzoites may rapidly traverse the Caco-2 cell layer within the lumens (Fig. 59A). To ensure the observed transmigration behavior reflects realistic biological processes *in vivo* and to confirm that these observations are not artifacts of the MPS system but reflect true bradyzoite behavior, we isolated mouse jejunal villi and infected them with *in vitro*-differentiated EGS bradyzoites. Our observational results showed that bradyzoites localize within the stroma in villi after 6 or 24 h post-infection (Fig. 5B i and ii). We also observed bradyzoites migrating between enterocytes, indicating that the parasites may reach the stroma without necessarily infecting the enterocytes themselves (Fig. 5B iii and iv).

To avoid the structural villi damage when scraping previously discussed (Fig. 1 and Fig. S12), we opted to use fixed villi prior bradyzoite infection. In fixed villi, we detected bradyzoites beneath the enterocytes after 6 h post-infection (Fig. 5C i) and an active bradyzoite internalization into the stroma (Fig. 5C ii). As expected, we did not see bradyzoites within the cells, indicating that they cannot invade the fixed tissue. Consistent with observations in unfixed villi (Fig. 5B iii), we found bradyzoites adjacent to areas of open enterocytes (Fig. 5C iii), suggesting a process of transient aperture of the enterocytes to reach the stroma (Fig. 59 iv).”

Figure 10. This is a very elegant way to look at this transmigration and infection of an adjacent MPS. For 10B was the seeding density very different to Figure 8 as there seem to be vastly different numbers of parasites and few to no bradyzoites on day 3 post infection in figure 8, whereas both stages are clearly visible in Figure 10, a lot of the red signal for tachyzoites expressing mCherry also looks artefactual, better representative images might be needed. There are structures in 10B zoom in that don’t appear in the lower resolution image (the red channel mostly, right hand side).

We appreciate the reviewer’s comments and suggestions. Indeed, we used different parasite densities for two different assays. We used 3 x 10^4^ bradyzoites for analyzing the switching dynamics in MPD shown in Figure 8 (now figure 4). While we used 5 x 10^4^ bradyzoites to study the migratory pattern of tachyzoites in MPD shown in Figure 10 (now figure 6). We cannot suggest if higher parasite density would improve switching, and this aspect should be addressed in future studies.

Reducing parasite density helps minimize GFP signal from dead bradyzoites in our switching assays in lumens. In contrast, we increased the parasite density for the migration assays in lumens to enhance the likelihood of observing any behavioral differences. The number of parasites used for infection has been updated in the legend for figure 6. Now it reads: *“Lumens were infected with 5 x 10^4^ bradyzoites of the EGS LDH2p-GFP/SAG1p-mCherry strain”*

In methods for figure 4 it now says: *“For the quantification of tachyzoite conversion, a total of seven plates, each containing at least three Caco-2 lumens, were infected with 3 x 10^4^ in vitro bradyzoites”.*

The prominent red area in the lumens shown in Figure 10C and D (now figure 6) resulted from foci of infection in a different plane than the free parasites in the matrix, which were not captured properly. Similarly, the discrepancy between the parasites in the magnified photograph in Figure 10B (now figure 6) and those in the unmagnified photograph is due to the difference in planes during image capture. The description of both events was incorporated in the figure legend of Figure 10 (now figure 6). It reads: “*Magnified photograph in (B) shows different parasites captured in different planes than those in unmagnified photograph”*.

10C the tachyzoites migrating through the matrix and to the other uninfected lumen is fantastic, it does appear that there are parasites visible by brightfield that don’t show up as mCherry positive, is this an imaging issue (not bright enough) or are these still bradyzoites or intermediates? It is convincing that these parasites migrate through the matrix and into the uninfected lumen. It looks like this occurs when an enormous number of parasites are found in one region (based on this one picture) is this a prerequisite or do parasites migrate into the matrix even if the density within the CACO2 cells it low?

We thank the reviewer for this observation. We agree that there is a population of parasites that are not fluorescent in either green or red, and we suggest that this may be an imaging issue. Regarding the high density of parasites, we do not know whether it improves or enhances parasite migration through the matrix; however, we agree that parasite density could lead to an interesting phenotype: it is possible that the increased density results in greater secretion of parasite components, which may enhance their ability to migrate through the matrix. This will be investigated in detail in a subsequent manuscript.

Reviewer #2: The IVIS experiments are very interesting and, in general, support previous observations suggesting infection is more common in the jejunal region of the intestine.

The migration experiments are clearly presented and will be of interest to the scientific community as will the cell culture system used (MPS)

The observation of the value of low temperature 33C on stage conversion is important as this may provide an improved way of obtaining in vitro cysts for both infection and other studies on this life cycle stage. Do you have any data on the gene expression in these low temperature cysts compared to those generated at 37C (e.g. RNAseq or scRNAseq)? Providing such data would be useful and interesting with regards to any differences that exist. Another alternative would be to look for RNA expression of genes only seen in cysts in vivo such as BCP1 (PMC5161674).

We appreciate Reviewer #2’s comments and suggestions. Unfortunately, we do not have data on the gene expression of 33°C- vs. 37°C-generated in vitro cysts. We agree that such data would be useful and interesting and we are planning and RNAseq experiment in the future that will compared 33°C- vs. 37°C-generated as well in vivo cysts.

Specific Comments

Line 191. Please expand why actin staining was not useful (usually actin epitopes can be seen in paraffin material treated in the manor described in your methods section).

We incorporated more details about actin staining in the results and methodology sections. The results now read: *“…as actin staining, which worked well for fresh or fixed villi, was ineffective for paraffin-embedded intestinal sections, possibly due to the long dehydration time during sample processing”.*

Line 252. I doubt the GFP cysts are expelled from host cells, but more likely host cells detach from the substrate and then are floating. A way to address this would be if the cysts have both a host cell membrane and cyst wall (TEM of the free floating cysts would demonstrate that 2 membranes, host cell plasma membrane and parasitophorous vacuole membrane surround the cyst.

We thank reviewer #2 for this clarification. We agree that the floating structures could be detached cells from the substrate. We have modified the text to describe both possibilities. Now it reads: *“After 9 days in differentiation media, we detected free floating GFP-positive cyst structures, possibly due to host cells detaching from the substrate or cyst structures being expelled from the host cells”.*

Line 275 to 285. IVIS in an intact animal really needs red shifted fluorescence, to that end it is not surprising that GFP was not detectable from the normal tissue signal and mCherry is not an ideal red shifted fluorochrome for this technique either in intact mice. Furthermore, the use of Alfalfa-free mouse chow is needed to decrease background fluorescence (is this what is referred to on line 283 as specialized diet) and is usually done for 7 day to make sure the gut is free anything that would cause autofluorescence.

Thank you for this information. All our mice are daily fed with alfalfa-free chow. Additional assays when standardizing included feeding with sunflower seeds and the special food Purified Rodent Diet (AIN-93G) to minimize autofluorescence. We did refer to this food in the text in the legend of the old supplementary figure 6. We decided to remove this data, given that it does not add much to the manuscript and distract from the exciting results.

Line 482-483 Why would the use of gut microorganisms be useful? Is there any data on a role for this in other pathogens and differentiation?

Few studies have investigated *Toxoplasma* co-infections. One study found that co-infection with *Toxoplasma* and *Eimeria* led to a reduction in macrophage phagocytosis of *Toxoplasma*. This co-infection appears to occur frequently in nature, particularly in chickens (PMID: 28983740). Co-infections with other parasites, such as *Schistosoma*, *Giardia*, or *Entamoeba histolytica* are also possible, especially in regions with high parasitic prevalence. The immunological impact of these co-infections is not yet fully understood but may involve complex interactions between the pathogens, potentially altering immune responses and disease outcomes. It remains unclear whether the presence of multiple organisms in the same environment as *T. gondii* could influence the parasite’s virulence, such as its motility, switching behavior, or other factors. As the MPS offers a platform for co-culturing organisms in controlled environments, there use could be a promising area for future research.

We incorporated more details in discussion, now it reads: *“These models offer valuable insights into stage conversion and infection progression, complementing traditional in vivo approaches. Additionally, co-infections with parasites like Schistosoma, Giardia, or Entamoeba histolytica—common in high-parasitic regions—may influence T. gondii virulence. The complex interactions between these pathogens and the immune response could impact T. gondii motility, switching behavior, and disease outcomes, making co-infection studies in MPS models crucial for understanding these dynamics”.*

Line 582 to 595 I agree that direct oral gavage makes sense, in that putting cysts into the mouth on bread might allow infection in the oral cavity without the cysts getting into the intestine. To that end, why is the positive control by gavage and the experimental condition by feeding on bread? Should not the exact same route been used for both the control and experimental group?

We thank Reviewer #2 for their comments. In our experiments, we used mice infected by consuming cysts in bread, as this route closely mimics the natural infection process. The oral gavage route was initially used to ensure direct delivery of cysts to the digestive tract. However, we agree with the comment and understand the confusion that may arise from comparing these two infection methods, and as a result, we removed the data related to the oral gavage route.

Line 653-655: Please provide the source for the various antibodies used (SAG1 rabbit, BAG1 rabbit, T. gondii polyclonal rabbit, CD31 rat and CD45 rat.

We apologize for not including this information in the first submission. We have updated the information regarding the antibody suppliers in the Methods section.

**Conclusions**

Reviewer #1: With the technical limitations, low numbers of sample and minimal quantification I find the statements in the discussion to be overreaching, especially when talking about the data from in vivo samples. Pepsin activation did not appear essential for in vitro culture in CACO2 cells in monolayer format, only in MPS. This is not adequately discussed.
The Discussion however is quite reflective of the observations using the MPS for stage transition kinetics and migration.

We would like to thank Reviewer #1 for their valuable comments and suggestions. We have made every effort to address them to the best of our ability. We added the missing information from the previous version and included new results to answer the raised questions and further support the observations made in this study. Additionally, we revised the language throughout the text to avoid overstatements.

Reviewer #2: The data are well organized and support the discussion and analysis.

Response: We thank Reviewer #2 for their positive feedback.

**Editorial and Data Presentation Modifications?**

Reviewer #1: The manuscript needs a thorough editing for which data should be included in the main body and what should be in the supplements. At present the key messages of the work are lost because all data relating to the project seem to be included in the main document with duplications in the supplement.
In addition there are grammatical and language issues throughout and a specialist edit for this after reformatting may be needed.

Our editions to improve the readability of the manuscript for lectors included: reorganized presentation of the first six figures into two main figures, we revised the number of mice for survival curves, and IVIS experiments. We included the number of intestinal samples analyzed and its corresponding quantification. We removed unnecessary information/figures in concordance with reviewers’ comments. We revised grammatical and language issues after reformatting.

Reviewer #2: None

**Summary and General Comments**

Reviewer #1: There is some exciting data presented in this work using novel technologies to give insight into the earliest interactions between T. gondii and the gut. The manuscript, however, lacks a clear narrative and suffers from over inclusion of optimization data, duplications of observational data and a lack of quantification and experimental details making the broader interpretation of the data difficult, especially in the context of the title and goals of the work.
I believe that the underlying data is of good quality, but a thorough reformatting of the manuscript is required. Frequently the supplementary data doesn’t show anything different to the figure in the main text, just provides an additional example of the same data which I don’t think is necessary and makes the narrative harder to follow.
I have tried to outline the areas where the manuscript narrative could be altered for clarity. The two required experiments are also highlighted with (Exp). All others, I believe, can be addressed by providing additional details and within the writing of the manuscript.

Reviewer #2: A very interesting paper with lots of data that will be of interest to people studying Toxoplasma differentiation and the early stages of infection via the oral route of infection.
